# Machine learning–driven decoding of maternal immune signatures in repeated pregnancy loss

**DOI:** 10.1371/journal.pcbi.1014806

**Published:** 2026-09-17

**Authors:** Tae Lyun Ko, Jaesub Park, Dongju Leem, Junho Kim, Jae won Han, Jin Sol Park, Sung Ki Lee, Hyojung Paik

**Affiliations:** 1 Center for Biomedical Computing, Korea Institute of Science and Technology Information (KISTI), and Department of Data and HPC Science, Korea National University of Science and Technology (UST), Daejeon, Republic of Korea; 2 Department of Bio and Brain Engineering, Korea Advanced Institute of Science and Technology, Daejeon, Republic of Korea, and Cambridge Stem Cell Institute, University of Cambridge, Cambridge, United Kingdom; 3 Department of Obstetrics and Gynecology, College of Medicine, Myunggok Medical Research Institute, Konyang University, Daejeon, Republic of Korea; 4 Department of Biological Sciences, Sungkyunkwan University, Suwon, Republic of Korea; Yale University, UNITED STATES OF AMERICA

## Abstract

Repeated pregnancy loss (RPL) is a multifactorial condition in which the underlying immunological mechanisms, particularly the disruption of maternal-fetal tolerance, remain incompletely understood. Although immune tolerance is critical for pregnancy success, the specific immune dysregulations contributing to RPL, particularly in euploid pregnancies, have been difficult to characterize. To address this, we performed single-cell RNA sequencing of decidual tissues from RPL patients and first-trimester controls. Our analysis initially revealed elevated expression of a transcriptional module of immune activation genes in RPL decidual tissues. To dissect the cellular drivers of this complex landscape, we employed genotype-based origin analysis coupled with a supervised machine learning model and a transformer-based foundation model (scGPT). This hierarchical approach prioritized maternal T cells over other immune subsets as the population carrying the most distinct and generalizable RPL-associated signatures. Through the convergence of computational drug repurposing, network centrality analysis, and a rigorous origin-controlled expression filtering strategy, we identified CXCR4 and JUN as druggable molecular candidates strongly associated with this T-cell dysregulation. Collectively, our machine learning–driven approach characterizes the maternal immune landscape of euploid RPL in the context of immune tolerance breakdown, and nominates candidate targets for future functional investigation.

## Author summary

Repeated Pregnancy Loss (RPL) is a devastating condition affecting many couples. It is particularly perplexing when it occurs even though the fetus is genetically normal, leaving doctors and patients without clear answers. Successful pregnancy requires the mother’s immune system to tolerate the fetus. However, the exact reasons why this tolerance fails in RPL have remained a mystery. In this study, we first found that in these unexplained miscarriages, maternal immune cells in the decidual tissue show increased activity of genes involved in immune activation. To characterize this immune activation in more detail, we applied advanced artificial intelligence to analyze the gene activity of individual cells at the interface between the mother and the fetus. This analysis identified maternal T cells as the immune cells most strongly and consistently linked to RPL. Furthermore, by mapping the complex molecular networks governing these cells, we identified specific molecules (CXCR4 and JUN) strongly associated with this immune dysregulation. This study provides a new way of understanding RPL in relation to immune tolerance failure and identifies these specific molecules as candidate targets for developing future treatments.

## Introduction

Repeated pregnancy loss (RPL) is defined as the loss of two or more consecutive pregnancies before 20 weeks of gestation [1]. While immune tolerance between the mother and the semi-allogeneic fetus is essential for successful pregnancy [2], the disruption of this tolerance has been proposed as a key driver of RPL [3]. However, the precise immune coordination at the maternal-fetal interface remains poorly understood, particularly regarding the distinct contributions of maternal versus fetal cells. To address this gap, we applied a machine learning-based approach to single-cell transcriptional profiles, aiming not only to prioritize cell types most strongly associated with RPL but also to identify the specific transcriptomic drivers underlying this immune dysregulation.

Clinically, RPL is heterogeneous, with causes ranging from anatomical defects to immunological dysfunction [4]. While fetal chromosomal aneuploidy is a known contributor, therapeutic interventions for such cases remain limited. Therefore, we focused on euploid RPL, where maternal etiologies—specifically immune dysregulation—play a dominant role [5]. Previous studies utilizing peripheral blood or bulk tissue analysis have provided valuable insights into immune tolerance failures [6]. To further elucidate the tissue-specific immune dynamics at the decidual interface, we sought to leverage single-cell resolution, which allows for a more granular dissection of cellular heterogeneity.

Single-cell RNA sequencing (scRNA-seq) offers a powerful solution for resolving this cellular heterogeneity. Comprehensive atlases have successfully mapped the healthy maternal-fetal interface [7,8]. Building upon these foundational resources, we aimed to distinguish the subtle, disease-associated transcriptional changes in RPL, a task that involves navigating the high dimensionality and complexity of the data. Conventional differential expression analysis may occasionally face challenges in fully characterizing complex, multivariate patterns essential for distinguishing pathological states [9]. This motivates the use of advanced computational techniques. Machine learning models can capture non-linear relationships [10], and recently adapted transformer-based architectures can highlight context-dependent gene relationships via attention mechanisms [11], providing new opportunities to decode the cryptic signatures of RPL.

In this study, we constructed a single-cell atlas of the human decidua comparing healthy pregnancies and euploid RPL cases. Beginning with an assessment of decidual immune activation, we subsequently employed a hierarchical machine learning framework and a transformer-based foundation model (scGPT) to identify key immune populations and robust transcriptomic drivers. This integrative computational framework enables a high-resolution dissection of decidual immune dysregulation associated with pregnancy loss.

## Results

### Patient cohort and dataset characterization

To characterize the transcriptomic landscape of euploid RPL, we collected first-trimester decidual biopsy specimens from women undergoing elective termination of uncomplicated pregnancies (Norm; n = 3; mean gestational age = 8.7 ± 1.5 weeks) and patients with RPL (n = 4; mean gestational age = 10.6 ± 2.5 weeks) (S1 Table). Given that fetal chromosomal abnormalities can significantly alter maternal immune responses and confound the computational analysis of immune-mediated mechanisms [12], we restricted our analysis strictly to cytogenetically normal cases. After excluding one RPL case identified with a chromosome 9 duplication (see Methods), the final cohort comprised matched maternal and fetal samples from euploid pregnancies. Matched peripheral blood samples were utilized to obtain maternal genotypes, which served as the ground truth for the subsequent computational demultiplexing of maternal versus fetal cells.

### Single-cell transcriptomic profiling and genotype-based demultiplexing reveal altered cellular landscapes in the RPL decidua

To comprehensively characterize the cellular heterogeneity of the maternal-fetal interface during early pregnancy, we performed single-cell RNA sequencing on decidual tissues. Following quality control, one normal case was excluded due to low sequencing quality, resulting in a final dataset of 62,588 single cells from two normal (Norm; mean gestational age = 9.5 ± 0.7 weeks) and three euploid RPL samples (mean gestational age = 10.3 ± 2.9 weeks) (see Methods). To determine the origins of cells as either maternal or fetal cells, we employed a dual-verification strategy, previously described in our study [13]. We first clustered cells based on allele variations using Souporcell [14] and validated these clusters against ground-truth genotypes from matched maternal PBMCs, utilizing an average of 927.3 ± 435.3 informative alleles per sample. This approach retained 57,976 cells with confidently inferred maternal or fetal origins for downstream analysis. Specifically, the final dataset comprised 35,027 (60.4%) cells from RPL samples and 22,949 (39.6%) from Norm samples; 41,517 (71.6%) were classified as maternal, and 16,459 (28.4%) were classified as fetal.

Next, we performed unsupervised clustering and cell-type annotation to resolve 32 distinct decidual cell clusters spanning immune and non-immune compartments. To independently verify the reference-based annotations, the expression of canonical marker genes for each cell type is shown in S1 Fig. These data were visualized using Uniform Manifold Approximation and Projection (UMAP) [15], colored by cell type, clinical condition (Norm vs. RPL), and inferred origin (maternal vs. fetal) (Fig 1A–1C). The inferred cell origins were successfully aligned with established biological knowledge: trophoblast lineages (EVT, SCT, VCT) and Hofbauer cells (HB) were overwhelmingly classified as fetal in origin; for instance, > 99.8% of EVTs were assigned as fetal (Fig 1E). This consistency reinforces the validity of our classification approach.

**Fig 1:**
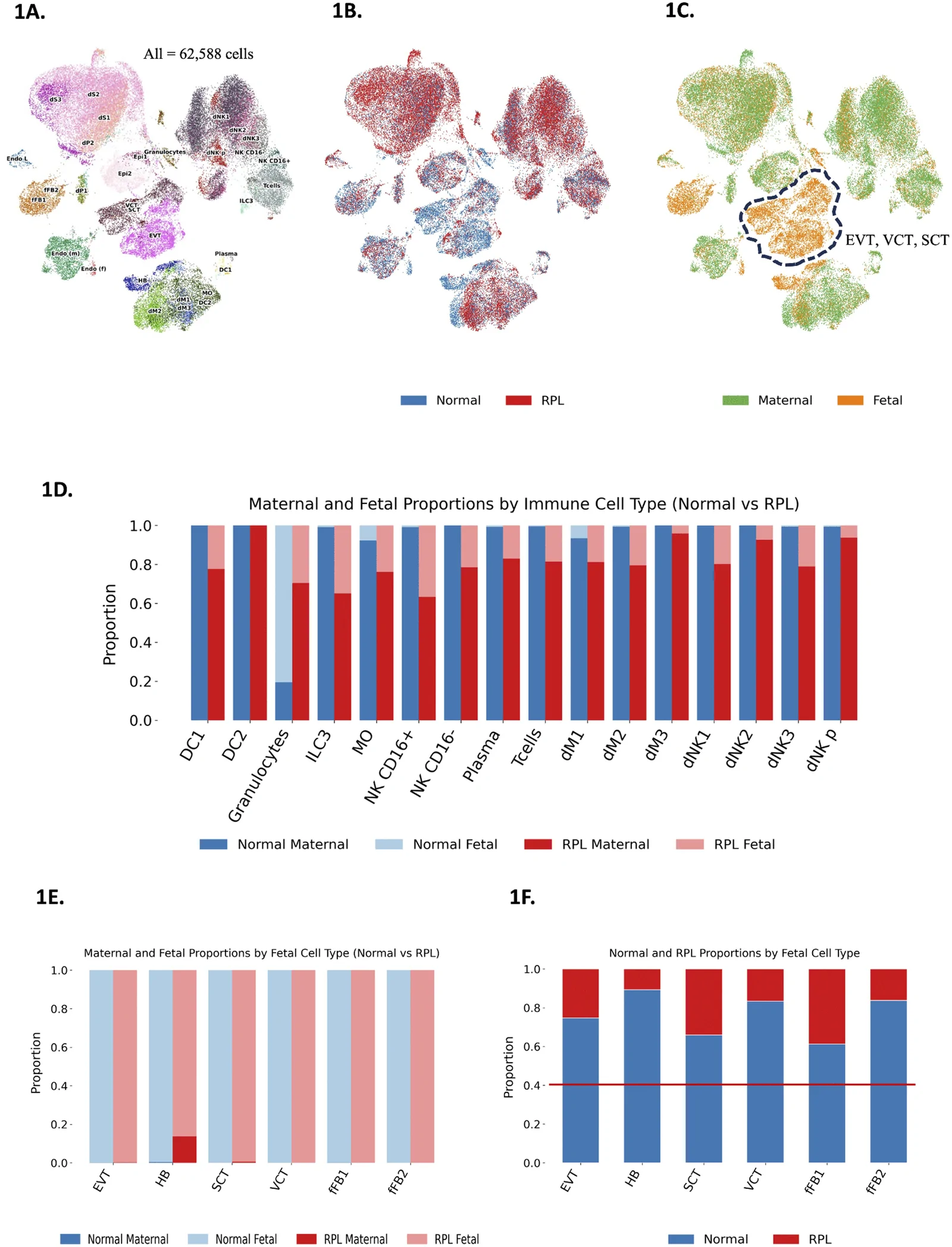
Single-cell landscape of the human decidua from elective terminations of normal pregnancies and RPL. (A) Uniform manifold approximation and projection (UMAP) embedding of cells from our study; each dot represents a single cell and is colored by cell type. Endo, endothelial cells; f, fetal; L, lymphatic; m, maternal; p, proliferative; Epi, epithelial glandular cells; SCT, syncytiotrophoblast; EVT, extravillous trophoblasts; VCT, villous cytotrophoblasts; DC, dendritic cells; dM, decidual macrophages; dS, decidual stromal cells; HB, Hofbauer cells; ILC, innate lymphoid cells; MO, monocytes; dNK, decidual natural killer cells; dP, decidual progenitor cells. (B) UMAP colored by clinical condition (Norm vs. RPL). (C) UMAP colored by inferred origin (maternal vs. fetal). (D) Stacked bar plots showing the proportions of maternal and fetal cells among immune cell types between Norm and RPL samples. (E) Stacked bar plots showing the proportions of maternal and fetal cells among fetal-derived cell types. (F) Stacked bar plots showing the proportions of cells from Norm and RPL samples among fetal-derived cell types. The red horizontal line indicates the expected baseline distribution based on the dataset composition (Normal: 0.4, RPL: 0.6).

Based on the validated cell origin classification, we next compared the composition of major cell types between the RPL and Norm samples. Most immune cell types, such as decidual natural killer (dNK) cells and T cells, were primarily maternal in normal cohorts (Fig 1D). Specifically, approximately 81% of NK cells across six subtypes and 80% of T cells were classified as maternal (S2 Table). However, we observed a notable proportion of fetal-origin immune cells in RPL cohorts. Conversely, trophoblast lineages and Hofbauer cells—both of fetal origin—exhibited lower proportions of RPL-derived cells than the baseline RPL proportion in the dataset (0.6) (Fig 1F). This relative underrepresentation may reflect differences in the cellular composition of RPL decidual tissue, though reduced fetal viability prior to tissue collection may also contribute.

### Distinct transcriptional landscapes and immune dysregulation in euploid versus aneuploid RPL

To assess the specificity of the immune impairment identified in our euploid cohort, we investigated whether the disease-associated transcriptional patterns were intrinsic to euploid RPL or shared with cases involving chromosomal abnormalities (aneuploid RPL). We employed the single-cell aneuploidy inference pipeline described in the Methods, which integrates dosage-sensitive gene expression (via scploid) and allelic imbalance at heterozygous SNP loci, to precisely identify fetal-derived decidual cells harboring chromosomal aberrations at the single-cell level within the samples included in this study. These identified cells were designated as the aneuploid subset, compared against euploid fetal cells, and were retained in all subsequent analyses.

We performed differentially expressed gene (DEG) analyses between RPL and Norm conditions separately within each subset to compare their regulatory landscapes (S2 Fig, S3 and S4 Tables). The analysis first revealed a set of immune-related genes that was consistently upregulated in RPL irrespective of ploidy. This group, which includes IL15, IGFBP1, IGFBP2, IGFBP7, and PRDM1, suggests a baseline level of stress-associated immune activation common to pregnancy loss [16–20]. However, the majority of signatures exhibited divergent regulation depending on chromosomal status. Key immune-activation genes associated with T/NK cell signaling (IL2RB, LCP2, ADGRE5, PTPRJ, PDE7A, RASA2) [21–27] were significantly upregulated specifically in euploid RPL but downregulated in aneuploid RPL. Conversely, genes related to mitochondrial metabolism and proteostasis (MRPL/MRPS subunits, COX5B, NDUFA11, VDAC1, PRDX3, PSMB5, CTSL) [28–33] exhibited the opposite trend, with strong upregulation restricted to aneuploid RPL. The biological distinction between immune-driven signatures in euploid cases and metabolic stress profiles in aneuploid samples highlights the distinct molecular pathologies of these conditions. These findings validate our strategy to specifically characterize the immune landscape within the euploid cohort, focusing on mechanisms distinct from the metabolic stress responses associated with aneuploid samples.

### Altered immune response signatures in maternal dNK cells

To obtain an initial readout of immune activation in the decidua, we began with the Common Rejection Module (CRM) [34], an 11-gene module defined by meta-analysis across multiple tissues to capture the signal of activated infiltrating immune cells rather than tissue-specific programs. Its constituent genes include interferon-γ–induced chemokines (CXCL9, CXCL10), IFN-inducible antigen-processing machinery (PSMB9, TAP1), T-cell surface and signaling molecules (LCK, CD6), and a cytotoxic granule gene (NKG7).

A comparison across all profiled decidual cells revealed that RPL samples exhibited significantly elevated CRM scores compared to Norm samples (Mann-Whitney U test, p = 1.27E-144; Fig 2A). To identify the specific cell populations manifesting this signature, we examined CRM scores at the cell-type level. Notably, decidual NK cell subsets dNK2 (p = 7.55E-13) and dNK3 (p = 1.23E-17) showed the most significant elevation in RPL, surpassing the mean CRM score across all immune cell types (immune baseline, 0.36; indicated as a dotted line in Fig 2B). Crucially, although we observed a notable proportion of fetal immune cells in RPL (Fig 1D), the dNK2 and dNK3 populations remain overwhelmingly maternal in origin. This indicates that the elevated activation signatures originate primarily from the maternal host. In healthy pregnancies, dNK2 and dNK3 are essential for recruiting extravillous trophoblasts and regulating invasion [35]. Furthermore, they actively modulate the local immune microenvironment through the secretion of cytokines and chemokines, such as XCL1, CCL3, and CCL4 [36]. However, their high CRM scores in RPL suggest a functional shift from these physiological roles.

**Fig 2:**
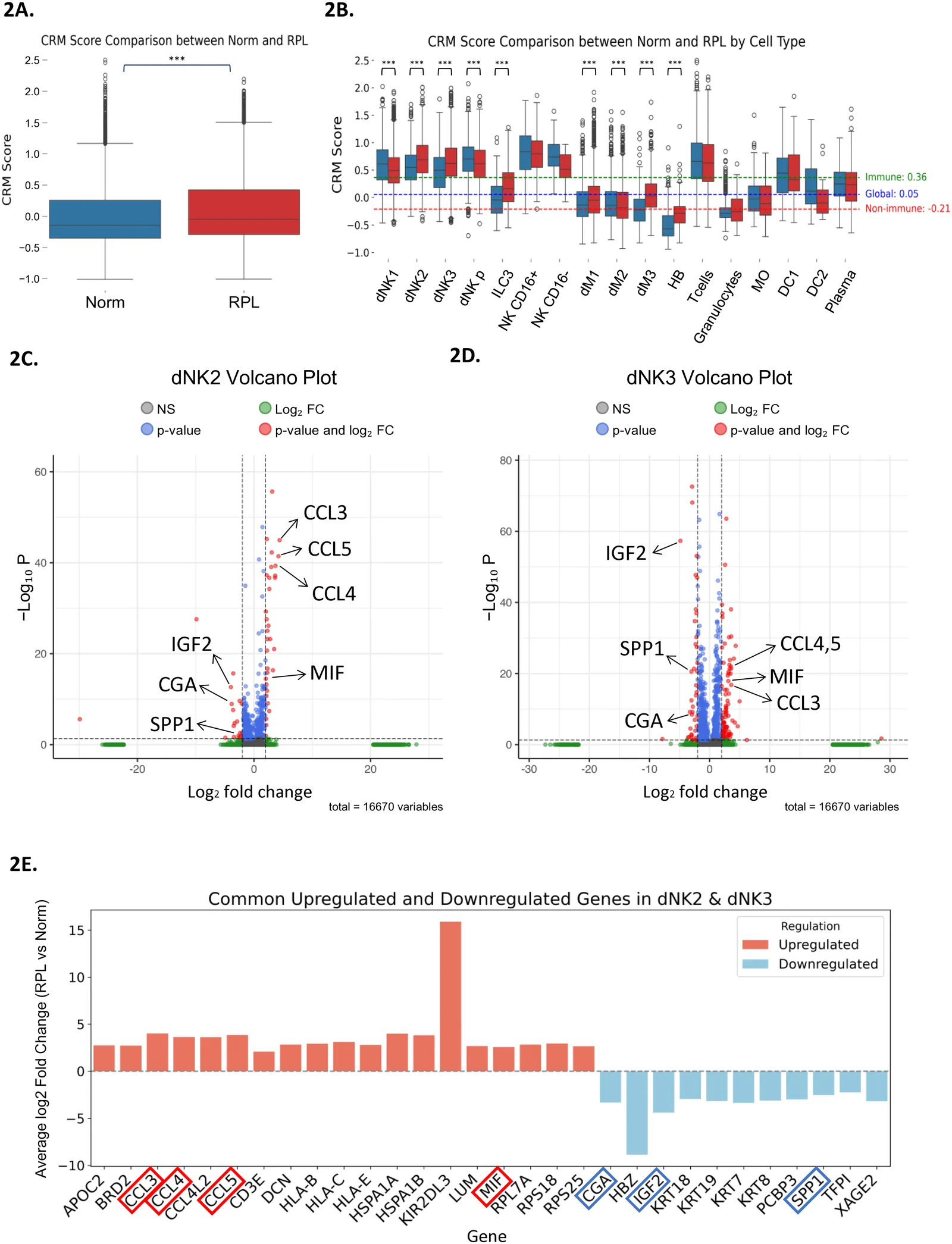
Altered immune response signatures in dNK cells from RPL patients. (A) Comparison of common rejection module (CRM) scores between Norm and RPL samples across all cells, showing elevated CRM scores in RPL. (B) Comparison of cell type-specific CRM scores between Norm and RPL samples. Among all immune cell types, dNK2 and dNK3 had the most significantly increased CRM scores in RPL, indicating heightened immune activation. The dotted lines indicate three reference baselines: the mean CRM score across all cells (global), across all immune cell types (immune), and across all non-immune cell types (non-immune). (C–D) Volcano plots showing genes that are differentially expressed between RPL and Norm samples in dNK2 (C) and dNK3 (D) cells. DEGs were defined as those whose adjusted p values were < 0.05 and absolute log2 fold changes were > 2. (E) Bar plot summarizing genes commonly upregulated and downregulated in both the dNK2 and dNK3 subtypes. Proinflammatory mediators (e.g., CCL3, CCL4, and MIF) were markedly upregulated, whereas immune tolerance–related genes (e.g., IGF2, CGA, and SPP1) were consistently downregulated, indicating immune dysregulation in RPL-associated dNK cells. Significance for the CRM score comparisons in (A) and (B) was assessed using the Mann-Whitney U test (*** p < 0.001), with Benjamini–Hochberg correction applied for the multiple cell-type comparisons in (B).

Guided by these scores, we performed DEG analysis within dNK2 and dNK3 to further elucidate the molecular basis of this shift (Fig 2C, 2D; S5 Table). To capture biologically robust transcriptional shifts, we specifically focused on genes exhibiting a greater than fourfold difference in expression levels between conditions. Using this criterion, we identified a consistent dysregulation pattern in both dNK2 and dNK3 (Fig 2E). Pro-inflammatory chemokines such as CCL3, CCL4, and CCL5 were upregulated, implying the recruitment of cytotoxic effectors (T cells and monocytes) via CCR5 signaling and the establishment of an inflammatory microenvironment [37]. The innate immune regulator MIF, known to amplify inflammatory responses and override glucocorticoid-mediated suppression, was also strongly induced [38]. In stark contrast, key tolerance-associated genes were suppressed. IGF2, a crucial growth factor promoting trophoblast development and fetal growth, was significantly downregulated [39]. Similarly, CGA, which encodes the alpha subunit of human chorionic gonadotropin (hCG) and plays a pivotal role in expanding regulatory T cells while suppressing NK cell cytotoxicity, was markedly reduced [40]. We also observed the downregulation of SPP1 (Osteopontin), a key cytokine that mediates cell-matrix interactions and promotes an anti-inflammatory Th2 shift at the maternal-fetal interface [41]. This concurrent upregulation of inflammatory mediators and downregulation of tolerance-inducing factors in maternal dNK cells provides transcriptomic evidence of the immune dysregulation characterizing RPL. A comparable analysis across all other immune cell types is provided in S3 Fig.

However, the marked disparity in maternal-to-fetal cell proportions between Norm and RPL samples (Fig 1D) acts as a critical confounding factor for standard statistical comparisons. Specifically, transcriptomic differences derived from population averages may predominantly reflect these compositional shifts rather than intrinsic disease-associated alterations. This limitation underscores the necessity for an alternative analytical strategy capable of disentangling true disease signatures from compositional biases.

### Hierarchical machine learning framework identifies RPL-associated immune cell populations

To overcome the limitations posed by cellular data imbalance and to capture multivariate disease signatures, we implemented a supervised machine learning framework. The primary purpose of this framework was not cell-type identification but rather the prioritization of genes distinguishing Norm from RPL states within each immune population. Unlike conventional statistical methods that test for mean differences in individual genes, this approach learns combinatorial transcriptomic patterns to classify cell states. Specifically, to effectively disentangle the robust transcriptomic features defining cell identity from the more subtle signals associated with the RPL condition, we employed a stepwise, hierarchical classification architecture implemented via devCellPy [42] (Fig 3A).

**Fig 3:**
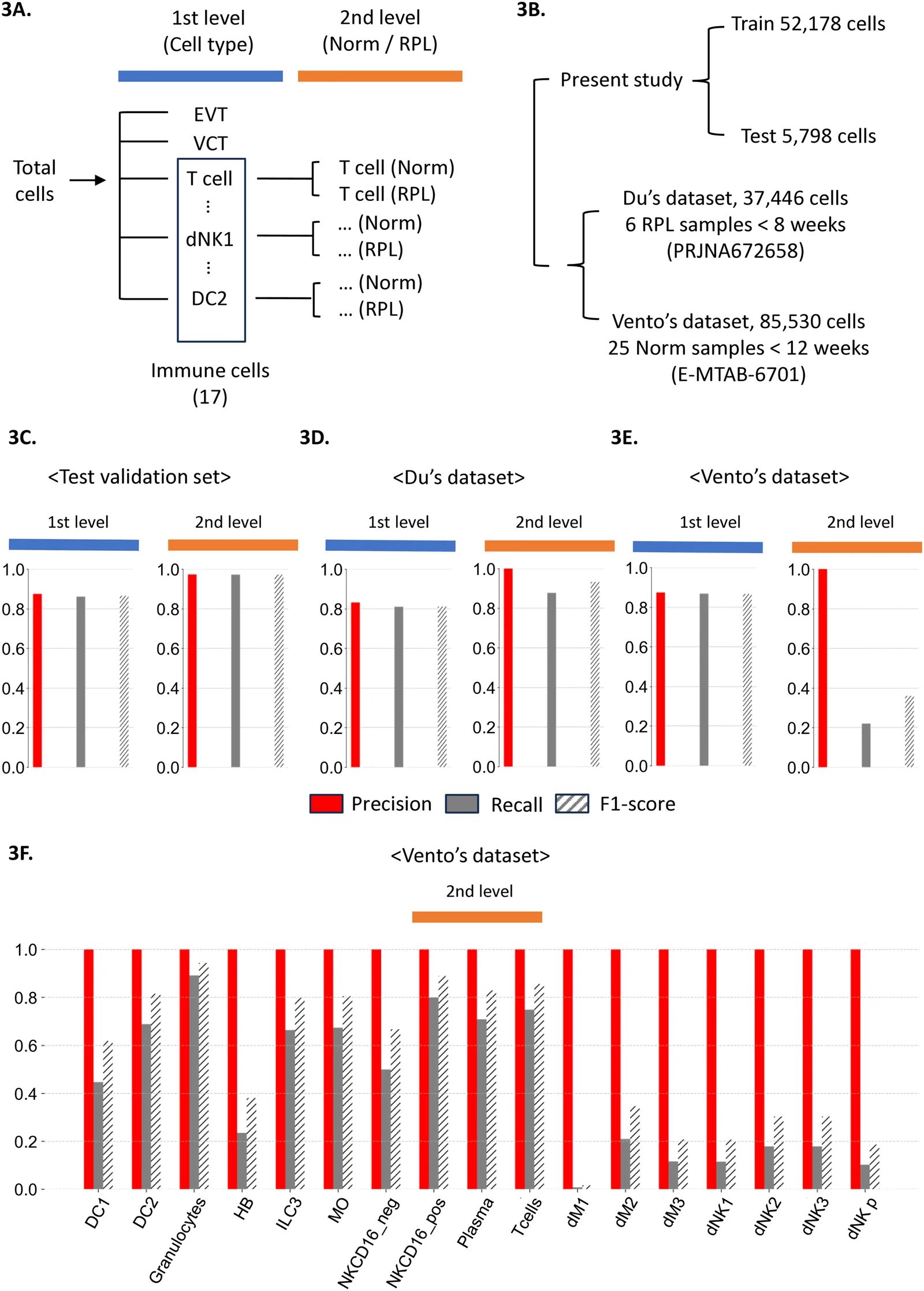
The machine learning approach revealed distinct immune cell signatures linked to RPL. (A) Schematic of the hierarchical classification framework using devCellPy. The model employs an XGBoost-based approach for multi-labeled, hierarchical cell identification. At the 1st level, cells are classified into 32 distinct lineage labels. Subsequently, at the 2nd level, the model distinguishes immune cell states based on pregnancy termination status (e.g., Tcells_Normal vs. Tcells_RPL). (B) Summary of the datasets used in this study. (C) Weighted average precision, recall, and F1 score across all cell labels in the held-out test set. The total number of classification labels in each level is also presented. The detailed performance of each cell label at each level is presented in S4 Fig. (D-E) Weighted average precision, recall, and F1 score for both 1st- and 2nd-level classifications across all cell labels in the Du (D) and Vento (E) datasets, respectively. (F) Weighted average precision, recall, and F1 score for each cell type at the 2nd level evaluated on Vento’s dataset. The performance at the 1st level is presented in S5 Fig.

The first level distinguishes 32 distinct cell identities based on lineage-specific signatures, while the second level classifies the identified immune cells into “Norm” or “RPL” states. For instance, T cells identified at the first level are further classified at the second level as T cells from Norm samples or T cells from RPL samples. A hypothetical third level distinguishing maternal versus fetal origins was excluded due to the biological scarcity of fetal-derived immune cells in normal controls (e.g., only 0.04% of Norm T cells were fetal), which precluded effective model training (S2 Table).

To evaluate the generalization capability of our model, we assessed its performance on a held-out test set and two independent external datasets selected to match our study’s biological context (Fig 3B). Du’s dataset [43] comprised 37,446 cells from six patients with recurrent miscarriage (5–8 weeks gestation; mean 6.83 ± 0.75 weeks). Crucially, these patients had ≥ 2 unexplained miscarriages and confirmed euploid karyotypes (no chromosomal abnormalities), ensuring alignment with our study’s focus on euploid RPL. Vento’s dataset [7] served as a healthy control benchmark, comprising 85,350 cells from uncomplicated elective terminations (6–12 weeks). These external datasets served as rigorous benchmarks to test whether the classifier could maintain predictive accuracy across distinct biological contexts and technical batches.

Performance metrics validated the efficacy of our hierarchical framework in resolving cell identities across diverse datasets and capturing RPL-associated patterns. In the internal test set, the model achieved high weighted average F1 scores at both first level (0.87) and second level (0.97) (Fig 3C), with high predictive accuracy maintained across the majority of individual cell types at both classification levels (S4 Fig). This high performance was replicated in the external RPL cohort (Du’s dataset), where the model achieved F1 scores of 0.81 (first level) and 0.93 (second level) (Fig 3D), again demonstrating consistent classification across most cell lineages (S5 Fig). These results confirm the model’s sensitivity in distinguishing RPL-associated transcriptomic states. In the external normal cohort (Vento’s dataset), while first-level classification remained robust (F1 = 0.87), second-level performance showed variability (F1 = 0.32) (Fig 3E), likely reflecting technical batch effects or the inherent challenge of applying models trained to capture disease signatures to exclusively healthy control datasets.

Despite these variations, T cells, granulocytes, and NK CD16 ⁺ cells were the only immune populations that retained high second-level classification performance in both external datasets (weighted average F1 scores of 0.86, 0.94, and 0.89 in Vento; Fig 3F). Although several other subsets achieved comparable or higher recall in Du’s dataset, their performance was not maintained in Vento’s dataset. These results indicate that these populations retain the most reliable discrimination between Norm and RPL states across differing clinical contexts, capturing RPL signatures in the RPL cohort (Du) while correctly classifying healthy cells in the normal cohort (Vento). Consequently, our hierarchical approach prioritized these cell populations as the key immune populations characterizing the RPL immune landscape, providing a filtered focus for subsequent gene-level investigations.

### Identification and cross-architecture validation of RPL immune signatures using transformer-based AI

To identify the specific genetic drivers underpinning our classification model, we performed level-specific Shapley Additive Explanations (SHAP) analysis [44]. This method quantifies the contribution of individual genes to the model’s decision-making at each layer. For each gene, a feature importance value was computed as the mean absolute SHAP value across cells. A higher value indicates a greater contribution to distinguishing the classes. We therefore ranked genes by these values to identify the strongest contributors to each classification. As a proof of concept, applying SHAP to the first level (cell identity) correctly identified canonical markers as top contributors—for instance, KRT18 for villous cytotrophoblasts (VCT) [45]. This confirmation of biological validity justified extending our analysis to the second level to isolate genes distinguishing Norm from RPL states within immune subsets. The complete results of the SHAP analysis for each cell type and level, including the SHAP values for individual genes, are presented in S6 and S7 Tables.

To validate these SHAP-derived signatures and rule out potential overfitting to the devCellPy hierarchical framework, we employed scGPT, a transformer-based foundation model pre-trained on large-scale single-cell transcriptomes [46]. Here, scGPT was used not to discover or define novel immune cell states, but as an independent, cross-architecture validation of the SHAP-derived signatures. This validation focused on the three cell types whose classification performance remained robust across both external datasets: T cells, granulocytes, and NK CD16 ⁺ cells. We hypothesized that if the top SHAP-ranked genes identified from these cell types represent true biological signals rather than model-specific artifacts, they should enable high classification accuracy even in a fundamentally different model architecture.

For each target cell type, we selected the top 100 SHAP-ranked genes from each of the two classification levels. These gene sets were introduced sequentially during fine-tuning, mirroring the two-level hierarchy: the first-level genes (cell identity) in the first fine-tuning step and the second-level genes (Norm vs. RPL) in the second (Fig 4A). We refer specifically to the second-level genes as the “RPL signature,” as these are the genes that distinguish the disease state. Using only these selected genes as input, the fine-tuned scGPT model performed a unified, single-step classification into six distinct states (Tcell_Normal, Tcell_RPL, Granulocyte_Normal, Granulocyte_RPL, NK_CD16 ⁺ _Normal, and NK_CD16 ⁺ _RPL).

**Fig 4:**
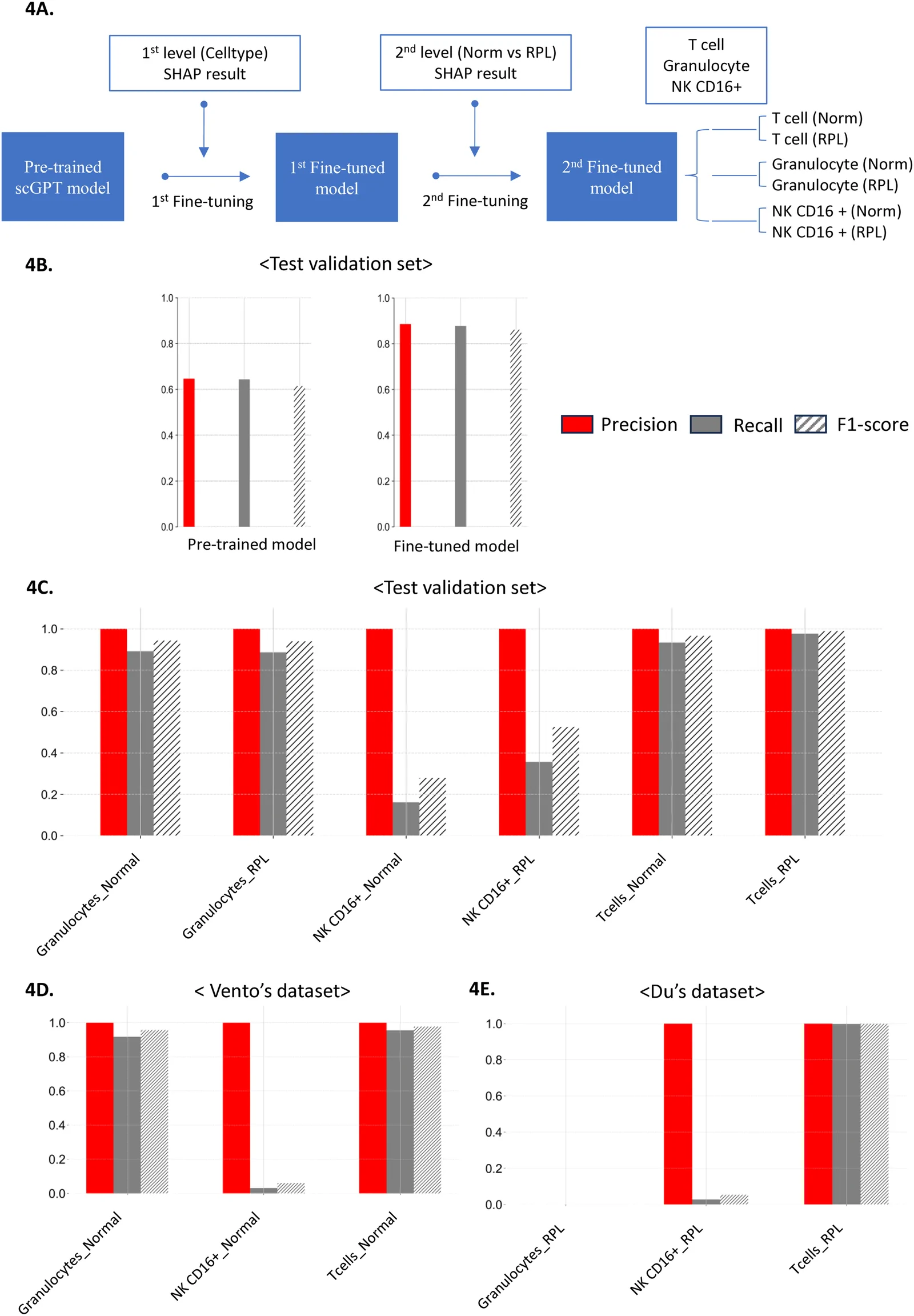
Fine-tuning the scGPT with SHAP-derived gene signatures robustly distinguished the RPL-associated immune cell subtypes. (A) Schematic of the fine-tuning strategy using the transformer-based scGPT_human model. The model was fine-tuned on the top SHAP-ranked genes from both classification levels for T cells, granulocytes, and NK CD16 ⁺ cells, introduced sequentially across two fine-tuning steps. Fine-tuning was performed on expression values restricted to this gene set. (B–E) Model performance was evaluated using the weighted average precision, recall, and F1 score. All performance results shown are for the final (second) fine-tuned model depicted in (A), which classifies cells into the six distinct states. (B) Comparison between the pre-trained scGPT model and the final fine-tuned model on the test validation set. (C) Performance of the final fine-tuned model on the test validation set for each immune cell subtype. (D) Performance of the final fine-tuned model on the independent Norm validation set. (E) Performance of the final fine-tuned model on the independent RPL validation set.

The results demonstrated the robustness of these signatures across model architectures. While the pre-trained scGPT model (zero-shot) achieved a baseline weighted F1 score of 0.61, fine-tuning with our identified signature genes improved performance to 0.86 (Fig 4B). This improvement confirms that the selected gene set contains robust and discriminatory information for RPL classification. Cell-type specific performance was particularly high; on the test set, T cells and granulocytes achieved high F1 scores (>0.94), whereas NK CD16 ⁺ cells showed distinctly lower recall compared to other cell types (Fig 4C).

We further validated the model on external datasets representing distinct biological conditions. In the Vento dataset (healthy controls), the model accurately classified T cells and granulocytes into their respective “Normal” subsets without misclassification, achieving high F1 scores (>0.96) (Fig 4D). Consistent with the internal test results, however, NK CD16 ⁺ cells exhibited poor generalization performance in this external cohort. This trend persisted in the Du dataset (RPL patients); while T cells maintained a weighted average F1 score of 0.99, NK CD16 ⁺ cells again failed to maintain robust classification accuracy (Fig 4E). Furthermore, granulocyte validation in the Du dataset was hindered by extreme sample scarcity (n = 9).

The observed results illustrate distinct validation outcomes for each cell type. While granulocytes showed high accuracy in healthy controls, their validation in RPL samples was limited by cellular scarcity. Conversely, NK CD16 ⁺ cells failed to generalize across external datasets. Therefore, T cells uniquely exhibited sustained high predictive accuracy across all validation cohorts, overcoming both the sample limitations and generalization challenges observed in other lineages. This consistent performance of T-cell-derived RPL signatures across distinct classification frameworks confirms their predictive power and highlights their potential for clinical diagnostic applications in RPL compared to other immune subsets. Given this robustness, we focused the subsequent functional and network analyses on the T-cell RPL signature, defined as the top 100 SHAP-ranked genes from the second-level (Norm vs. RPL) classification of T cells.

### Integrative pathway and network analyses of SHAP-derived RPL signatures reveal immune-associated mechanisms and therapeutic targets of RPL

We assessed the potential clinical relevance of this T-cell RPL signature. Overrepresentation analysis of the signature revealed enriched cellular functions, with pathways showing adjusted *P*  <  0.05, which were considered significant. The top 20 pathways were ranked by odds ratio, defined as the proportion of query genes mapped to each pathway. This analysis revealed marked enrichment of RPL signatures in five biological categories: translation machinery, mRNA quality control, viral translation, ROBO signaling, and nutrient stress response (Fig 5A, S8 Table).

**Fig 5:**
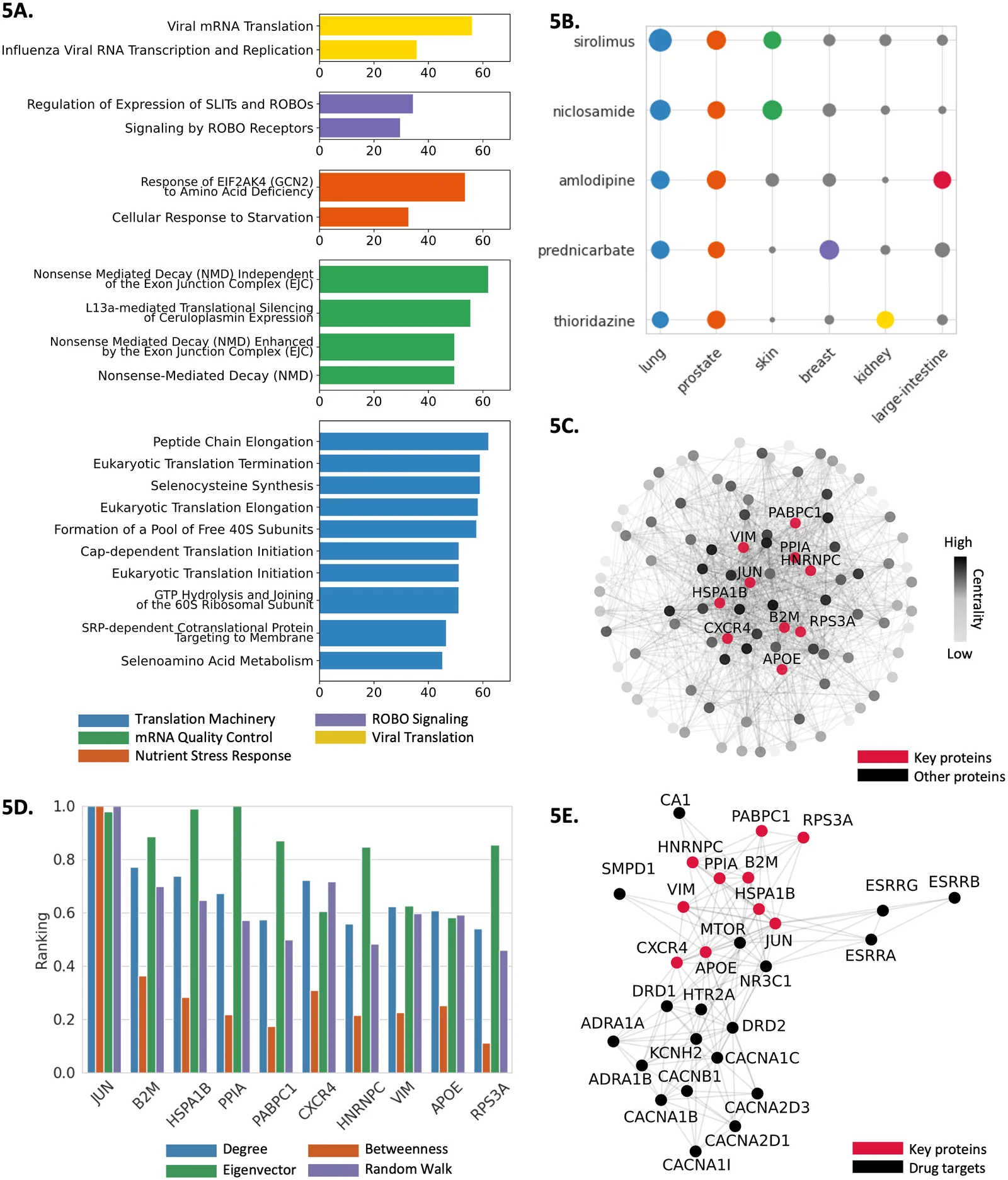
Pathway analyses and drug repurposing reveal clinical implications of T-cell signature genes in RPL. (A) Bar plot showing the results of overrepresentation analysis (ORA) of 100 RPL signatures using the Reactome pathway database. The top 20 pathways with the highest odds ratios, among those with adjusted P values ≤ 0.05, are displayed. Pathways are grouped according to functional categories. (B) Dot plot showing drug repurposing predictions for RPL based on RPL signatures using ASGARD. Each dot represents a predicted drug, with the dot size inversely proportional to the P value. Dots corresponding to drugs with P values ≤ 0.05 are color-coded according to the tissue type in which the prediction was made. (C) Network visualization of interactions between RPL signatures based on STRING protein–protein interaction data. Node color intensity reflects average centrality. The top 10 signature genes with the highest average centrality are labeled and highlighted in red. (D) Bar plot showing the centrality scores of the top 10 signatures in the protein–protein interaction network. Centrality values were min–max normalized to range between 0 and 1. (E) Visualization of the protein–protein interaction network of the top 10 RPL signatures and target proteins of the 5 repurposed drugs.

Among the five functional groups, translation machinery exhibited the greatest number of enriched pathways, which was consistent with the T-cell origin of the RPL signatures. Upon antigen stimulation, T cells exit quiescence and undergo extensive transcriptional and metabolic reprogramming, processes closely linked to translational control during activation and immune regulation [47–49]. The mRNA quality control group was predominantly associated with nonsense-mediated decay (NMD), a post-transcriptional mechanism increasingly recognized for its role in shaping immune responses and emerging as a therapeutic target in autoimmunity [50,51]. ROBO signaling, which is implicated in early trophoblast function, may contribute to RPL through placental dysregulation [52,53]. Although the connections of viral translation and nutrient stress response pathways to immune tolerance are less clearly defined, their relevance is supported by established links between pregnancy loss and immune responses [54–56]. Together, these findings underscore the strong immunological component of the RPL transcriptomic signatures.

We next asked whether reversing RPL-associated transcriptional changes could mitigate RPL pathology. To address this, we applied ASGARD [57], a drug-repurposing algorithm that matches disease-associated gene expression profiles with compounds from the L1000 drug-response dataset predicted to counteract those signatures (see Methods). ASGARD identified five candidate compounds predicted to significantly reverse RPL-associated gene expression in at least three distinct tissues (Fig 5B, S9 Table). All five drugs showed predicted efficacy in lung and prostate tissues, with variable activity across other tissues. Notably, three compounds (prednicarbate, sirolimus, and niclosamide) have previously been implicated in contexts relevant to RPL [58–65], providing orthogonal support for their therapeutic potential.

Corticosteroids such as prednicarbate are typically used to treat autoimmune conditions and are administered to women with repeated early pregnancy loss [58,59]. Recent meta-analyses report a significant increase in ongoing pregnancy rates following corticosteroid treatment [60]. Sirolimus (rapamycin), which inhibits T- and B-cell activation and proliferation, is approved for use in transplantation and oncology [61,62]. A recent phase II randomized trial revealed that sirolimus significantly improved both pregnancy and live birth rates in patients with repeated implantation failure [63]. Niclosamide has been proposed as a therapy for immune-mediated disorders, including autoimmune diseases and infections, through the modulation of immune pathways [64]. Notably, its inhibition of follicular helper T cells has been shown to be effective in preclinical autoimmune models [65]. Although neither thioridazine nor amlodipine has been directly associated with RPL or immune tolerance, calcium channel blockers such as amlodipine exhibit known immunosuppressive effects that may warrant further investigation [66,67]. These findings support the validity of our drug-repurposing predictions and further implicate RPL gene signatures in the pathophysiology of RPL.

To identify candidate therapeutic targets from the RPL gene signatures, we constructed a protein–protein interaction (PPI) network by mapping the T-cell RPL signature genes to their encoded proteins and assembling them using STRING (Fig 5C). In such networks, hub proteins with high connectivity are more likely to modulate overall network behavior [68,69]. To prioritize central nodes, we computed four network measures (degree centrality, betweenness centrality, eigenvector centrality, and random walk) and ranked the proteins based on their average centrality scores. The top ten hub proteins were selected from these 100 signature proteins (Fig 5D, S10 Table). These hub proteins exhibited dense connectivity with targets of repurposed drugs identified earlier (Fig 5E). In particular, mTOR is the molecular target of sirolimus, one of our top repurposing candidates. Its dense connectivity with the hub proteins links our network analysis to an independently predicted compound, and is further supported by mTOR’s established role in T-cell–mediated immunity and autoimmune disease.

A focused literature review revealed that seven of the ten top-ranked hub proteins are associated with RPL or immune regulation, further supporting their therapeutic potential. CXCR4, for instance, plays a critical role in coordinating both innate and adaptive immune responses [70]. Additionally, a recent systematic review and meta-analysis revealed a significant association between maternal APOE genotype and RPL risk in Asian populations [71]. Single-nucleotide polymorphism analysis involving more than 200 patients further suggested that APOE variants may predispose individuals to RPL [72]. Although JUN, HSPA1B, PPIA, and VIM have not been directly linked to RPL, these genes are functionally implicated in T-cell activity and activation and are known to contribute to immune modulation [73–79]. Together, these findings highlight the functional relevance of the identified RPL signature genes in the pathophysiology of RPL and support their potential as biomarkers and therapeutic targets for future translational studies.

### Origin-controlled expression filtering identifies robust T-cell–associated transcriptomic signatures in RPL

Building upon the insights from the functional and network-based characterization of SHAP-derived T-cell signatures, we next sought to further refine the top-ranked genes derived from the second-level classification (Norm vs. RPL). We evaluated whether their expression patterns remained consistent after accounting for potential confounding effects, such as differences in cell origin or cellular composition. To this end, we performed an origin-controlled expression filtering analysis aimed at identifying genes that exhibit disease-specific transcriptional changes independent of sample-level heterogeneity.

In particular, nearly all the Norm T cells were maternal in origin, whereas a substantial proportion of the RPL T cells were fetal in origin (S2 Table). This imbalance raised the possibility that some SHAP-ranked genes might reflect origin-related differences (Maternal vs. Fetal) rather than RPL-associated changes (Norm vs. RPL). To eliminate this confounding effect, we implemented a two-step validation approach (Fig 6A). First, we excluded genes whose expression levels significantly differed between maternal and fetal T cells. Next, from the remaining pool, we retained only those genes that showed significant differential expression between RPL and normal T cells strictly within the maternal subset.

**Fig 6:**
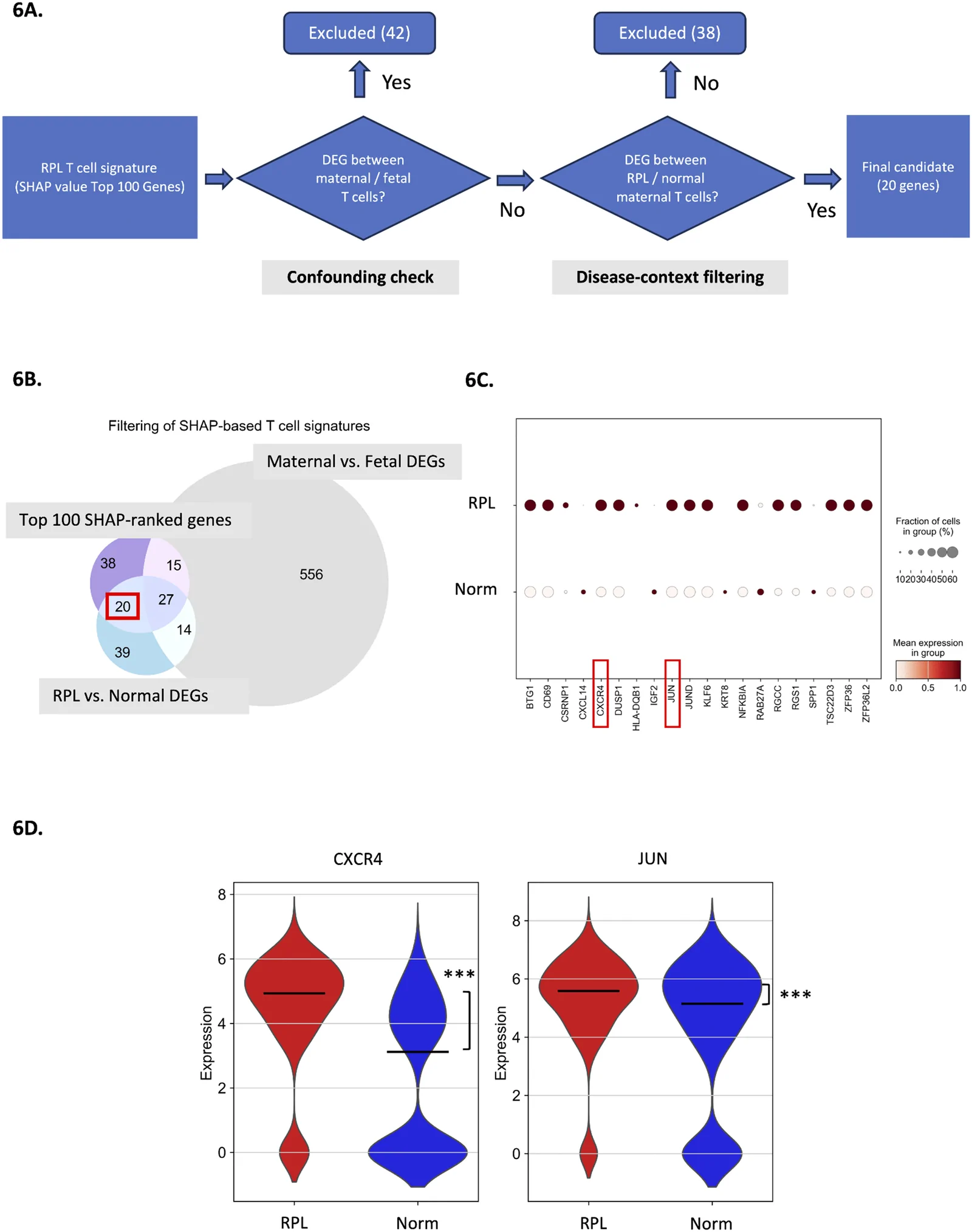
Confounding-controlled filtering identifies robust T-cell signatures in RPL. (A) Schematic of the two-step filtering strategy used to refine SHAP-ranked T-cell genes by removing potential confounding effects. Genes differentially expressed between maternal and fetal T cells (n = 42) were excluded to control for cell origin-associated bias (confounding check). Among the remaining genes, those that were not significantly differentially expressed between RPL and Norm maternal T cells (n = 38) were further excluded. This yielded a final panel of 20 genes showing RPL-specific expression patterns. (B) Venn diagram illustrating the overlap among the top 100 SHAP-ranked genes, maternal vs. fetal T-cell DEGs, and RPL vs. Norm maternal T-cell DEGs. The red-boxed sector (n = 20) represents genes included in the SHAP top set and RPL-related DEGs but not in maternal–fetal DEGs—comprising the final RPL-specific candidates. (C) Dot plot showing the expression and cellular prevalence of the 20 final candidate genes in RPL and Norm T cells. The dot size indicates the fraction of cells expressing the gene; the color indicates the average expression. All 20 genes were significantly differentially expressed between RPL and Norm maternal T cells. (D) Violin plots of CXCR4 and JUN expression in RPL vs. Norm maternal T cells. Both genes were retained after filtering and showed elevated expression in RPL T cells. Statistical significance in (C) and (D) was assessed using the Wilcoxon rank-sum test with Benjamini–Hochberg false discovery rate (FDR) correction (*** adjusted p < 0.001).

This filtering process distilled the initial candidate list (the top 100 SHAP-ranked T-cell genes, selected from the second-level SHAP results in S7 Table) down to a robust panel of 20 genes (Fig 6B), with 13 genes showing consistently elevated expression in RPL across wide cellular distributions (Fig 6C). These genes represent the candidates for RPL-specific immune markers, supported by a dual validation strategy: machine learning–based prioritization and disease-contextual expression filtering.

Significantly, this independent filtering process converged with our previous network findings. Among the 20 retained genes, CXCR4 and JUN were directly identified as top-ranked hub proteins in the preceding protein–protein interaction (PPI) network analysis (Fig 5D). This convergence between network centrality (potential for systemic regulation) and confounding-controlled expression (robustness against artifacts) strongly supports their biological relevance, highlighting them as the most reliable candidates implicated in RPL. The expression levels of both genes were significantly upregulated in RPL T cells compared to Norm cells (Fig 6D).

CXCR4 encodes a chemokine receptor that regulates leukocyte trafficking and inflammatory responses, particularly through the CXCL12–CXCR4 axis [80]. In normal pregnancy, CXCR4 promotes immune tolerance and tissue remodeling, including decidual NK cell migration [81,82]. However, dysregulated CXCR4 expression is linked to excessive T-cell infiltration and sustained inflammation in autoimmunity [83,84]. Similarly, JUN, a key component of the AP-1 transcription factor complex, integrates T-cell receptor (TCR) signals to activate T-cell programs [85,86]. Upon stimulation, JUN translocates to the nucleus and initiates chromatin remodeling and cytokine transcription [87,88]. While its role in normal pregnancy involves trophoblast invasion [89], JUN overexpression in pathological contexts can sustain inflammatory responses and prevent T-cell exhaustion, promoting persistent immune activation [74,90].

Together, these findings suggest that CXCR4 and JUN may contribute to pathological immune activation at the maternal–fetal interface in RPL. To our knowledge, neither gene has been previously highlighted as a distinguishing feature of decidual T cells in the context of RPL, underscoring their potential novelty and relevance as key immune signatures in pregnancy loss.

## Discussion

In this study, we employed single-cell RNA sequencing and advanced computational modeling to investigate the transcriptomic correlates of immune tolerance failure in euploid RPL. To ensure accurate cellular classification, we utilized genotype-based validation (Souporcell), which enabled the reliable distinction between maternal and fetal cells. This distinction revealed an altered cellular landscape in RPL, including a reduced proportion of fetal-derived trophoblasts alongside a higher proportion of fetal immune cells.

Our integrative analysis highlights a complex interplay between distinct immune subsets in RPL pathology. The elevated CRM scores are consistent with heightened immune activation in the RPL decidua. Building on this observation, our machine learning analysis prioritized T cells as the source of the most distinct and generalizable transcriptomic signatures for patient stratification.

To validate these T-cell gene signatures and confirm their robustness across internal validation and independent external cohorts, we employed a cross-architecture validation strategy using the transformer-based foundation model, scGPT. The successful transfer of signatures from a hierarchical classifier to a pre-trained transformer confirmed that these markers represent robust biological signals rather than model-specific artifacts. Furthermore, pathway and network analyses linked these signatures to translation machinery and identified CXCR4 and JUN as central hub regulators. Crucially, these two genes emerged from the convergence of independent network centrality analysis and strict origin-controlled expression filtering, reinforcing their validity as key candidates associated with T-cell dysregulation in RPL.

Despite these insights, several limitations warrant consideration. First, our study cohort was clinically heterogeneous and relatively small (Norm n = 2, RPL n = 3). While we leveraged the high-dimensional resolution of 62,588 single cells to capture fine-grained transcriptional states, the limited number of biological replicates necessitates cautious generalization. We attempted to mitigate this by rigorously validating our findings across multiple independent datasets (Du and Vento). However, the feasibility of rigorous cross-architecture validation was constrained by data availability for certain cell types; for instance, granulocyte validation in the Du dataset was limited by cellular scarcity, and NK CD16 + cells showed reduced generalization in transformer-based modeling compared to the initial machine learning results. In contrast, T cells uniquely maintained high predictive accuracy across all validation stages, further justifying our primary focus on T-cell signatures.

Second, the recorded gestational age differed between groups (Norm, 9.5 weeks; RPL, 10.3 weeks). We note that in RPL, the recorded gestational age reflects the time of clinical diagnosis rather than the actual timing of embryonic demise, as RPL frequently presents as a missed abortion in which a non-viable pregnancy is retained until detected at a later examination. Furthermore, both groups fall within the first trimester, during which the major decidual immune populations remain relatively stable [91]. Nevertheless, we acknowledge that gestational age represents a potential confounding variable that cannot be fully excluded in cross-sectional studies of this nature. Future studies with more strictly matched gestational ages would be valuable to further validate these findings.

Third, comparisons of cell-type composition between groups should be interpreted with particular caution, as single-cell proportions are sensitive to pre-analytical factors such as tissue sampling site, biopsy depth, and dissociation efficiency. In particular, the reduced representation of fetal-derived trophoblasts and Hofbauer cells in RPL samples may not reflect a decidua-intrinsic pathogenic mechanism, but could instead result from reduced fetal viability prior to tissue collection, which is common in RPL. Orthogonal validation, such as immunostaining-based quantification on standardized tissue sections, would be required to firmly establish such compositional differences. We therefore regard our compositional observations as contextual rather than definitive, and emphasize that the central conclusions of this study rest on the machine learning–derived transcriptomic signatures rather than on cell-proportion comparisons.

Finally, while CXCR4 and JUN emerged as robust markers, our findings establish association rather than causality. Nevertheless, their identification through the convergence of independent network analysis and origin-controlled filtering supports their reliability as candidate markers. Future studies incorporating functional perturbation (e.g., organoid models or in vivo edits) are essential to determine whether their upregulation is a primary driver of pregnancy loss or a secondary consequence of fetal demise or miscarriage-related pathology.

In conclusion, by integrating single-cell transcriptomics with complementary AI frameworks, we identified robust T-cell–associated signatures that distinguish euploid RPL from normal pregnancies. The convergence of machine learning prioritization and network analysis refined these targets, while in silico drug repurposing further highlighted the translational potential of our findings. These results lay the groundwork for developing precision biomarkers and targeted therapeutic interventions to improve the stratification and management of unexplained RPL.

## Methods

### Ethics statement

All participants provided written informed consent, and the study was approved by the Institutional Review Board (IRB) of Konyang University Hospital (IRB approval number: KYUH 2022-08-009-005).

### Sample collection

Decidual tissue (transformed endometrium during pregnancy) samples were collected from seven pregnant individuals, including three with normal pregnancies and four with RPL. In both groups, decidual tissue and peripheral blood were collected via dilation and curettage (D&C). Tissue was collected under ultrasound guidance, with sampling directed at the decidua basalis. Owing to the nature of the D&C procedure, a minor contribution from other decidual compartments cannot be fully excluded; however, the collected tissue consisted predominantly of decidua basalis. In the normal group, samples were obtained during the elective termination of pregnancy, whereas in the RPL group, two resulted from spontaneous miscarriage, and two were due to elective termination. To investigate immune tolerance failure in the absence of fetal chromosomal anomalies, we performed cytogenetic karyotyping on all samples. One RPL case exhibiting a chromosome 9 duplication was identified and excluded. Consequently, the remaining cohort consisted of three normal controls and three RPL cases, all of which were confirmed to have normal euploid karyotypes. One normal sample was subsequently excluded at the sequencing quality-control stage, so that the final analyzed cohort comprised two normal controls and three RPL cases. All the samples were collected under sterile conditions and immediately processed for further analysis, including single-cell RNA sequencing (scRNA-seq).

### Analysis of droplet-based single-cell RNA sequencing data

Single-cell suspensions were prepared through standard tissue dissociation and cell processing procedures. Single-cell RNA libraries were constructed using the 10X Genomics Chromium Platform in accordance with the manufacturer’s instructions. Libraries were sequenced using both the Illumina HiSeq-x and NovaSeq 6000 platforms. The raw sequencing reads were aligned to the reference genome (GRCh38) using Cell Ranger (v5.0.0) to generate gene‒cell count matrices. Ambient RNA noise was minimized using SoupX [92], and potential doublets were removed with Scrublet [93]. Preprocessing and quality control were conducted in Scanpy (v1.10.1), filtering out cells with mitochondrial gene expression exceeding 20%, fewer than 200 detected genes, fewer than 1,000 UMI counts, or more than 7,000 total genes per cell. These quality control steps yielded a final dataset of 62,588 high-quality cells for downstream analysis. The merged data underwent normalization, scaling, and the identification of highly variable genes, followed by dimensionality reduction using principal component analysis (PCA) and cluster visualization with UMAP [15]. Batch effects were corrected using Harmony [94] after the clustering results were compared with those of BBKNN [95]. Cell types were identified using a logistic regression modeling approach implemented in sceleto2 (https://pypi.org/project/sceleto2/), in conjunction with CellTypist [96], based on reference labels for cells in the human decidua during the first trimester [7].

### Identification of fetal and maternal cells

Given the nature of the samples, which were obtained from the decidua of pregnant women following pregnancy termination, the tissues contain a mixture of maternal and fetal cells. Therefore, to accurately determine whether the acquired cells originated from the mother or the fetus, we employed two independent analytical methods for cross-validation. This approach enabled us to reliably identify the origin of each cell. We first utilized Souporcell [14] to cluster the cells based on their genotypes, dividing them into two genetically distinct clusters. To verify the origin of each cluster, we subsequently compared the genotype profiles generated by Souporcell with the genetic variant data obtained from maternal peripheral blood mononuclear cells (PBMCs) using the Illumina GSA v3 genotyping array. The single-nucleotide polymorphisms (SNPs) observed in the two clusters were compared with the maternal PBMC genomic data to assess their similarity. The cluster with higher similarity was determined to consist of maternal cells and was defined as the “maternal cell cluster,” whereas the cluster with lower similarity was considered to consist of fetal cells. To achieve this goal, an average of 927.3 ± 435.3 alleles per sample were examined, allowing us to genetically distinguish the two clusters and identify the origin of each cell.

### Identification of single cells with aneuploidy from scRNA-seq data

Chromosomal aneuploidy was investigated in each cell of the scRNA-seq dataset using the method previously described by Starostik et al [97]. Briefly, chromosomal aneuploidy can be inferred by integrating two types of information: chromosome-wide differential expression of dosage-associated genes and allelic imbalance at heterozygous SNP sites. For each single cell, the expression levels of dosage-associated genes were quantified using scploid, which provides a predefined set of dosage-associated genes along with benchmarking data from mosaic aneuploid mouse embryos [98]. Allelic imbalance was estimated per chromosome by measuring allelic read counts at each heterozygous SNP site.

To apply this method to our scRNA-seq dataset, we first generated aligned BAM files for every single cell using a custom python script that split the BAM files by cell barcode. Based on cell cluster information obtained from Souporcell (see above), maternal and fetal pseudobulk BAM files were generated by merging cells using SAMtools (version 1.3.1) [99]. Heterozygous SNPs were identified from the pseudobulk using GATK HaplotypeCaller [100]. Allelic read counts were then measured for each heterozygous SNP site using GATK ASEReadCounter for every cell. Owing to the sparsity of 10X Chromium scRNA-seq data, we included only SNP sites with at least three mapped reads per cell. Based on the measured read counts, we computed the allelic ratio for each chromosome–cell pair by dividing the sum of minor allele reads by the total number of mapped reads. Aneuploidy was determined if the scploid-derived p value was ≤ 0.05 and if the allelic ratio was within the top 10% across all chromosome–cell pairs.

### Calculation of the common rejection module score

The common rejection module (CRM) score is calculated using the expression levels of 11 predefined CRM genes: BASP1, CD6, CXCL10, CXCL9, INPP5D, ISG20, LCK, NKG7, PSMB9, RUNX3, and TAP1 [34]. These genes were identified through meta-analyses as being consistently overexpressed during acute rejection (AR) across multiple transplanted organs. The score is derived by averaging the expression values of these CRM genes and subtracting the average expression of a dynamically selected set of control genes. This process ensures that the score reflects the relative activity of the module genes while accounting for background transcriptional noise. The calculations were performed using the Scanpy package. Statistical significance of CRM score differences between groups (e.g., Norm vs. RPL) was assessed using the Mann-Whitney U test, with Benjamini–Hochberg correction applied for multiple comparisons.

### Differential gene expression analysis and origin-controlled filtering

Differential gene expression analysis was performed using the Wilcoxon rank-sum test as implemented in scanpy.tl.rank_genes_groups, with Benjamini–Hochberg correction applied for multiple testing. All analyses were conducted on log-normalized gene expression values. Genes were considered differentially expressed if the adjusted *p* value was less than 0.05 and if the absolute log2 fold change exceeded 1. Comparisons were made between biological conditions of interest, including maternal versus fetal origin and RPL versus normal samples, depending on the analysis context.

To identify robust disease-specific signatures independent of sample-level heterogeneity (e.g., cell origin imbalance), we implemented an origin-controlled expression filtering strategy within this statistical framework. First, we excluded genes showing significant differential expression between maternal and fetal cells (confounding markers). Second, from the remaining genes, we retained only those that were significantly differentially expressed between RPL and Norm conditions strictly within the maternal cell subset. This two-step filtering ensured that the identified signatures reflected true pathological changes rather than artifacts arising from cell origin proportions.

Separately, for an analysis focused on the aneuploid cell subset, we additionally employed the DESeq2 package in R [101]. This approach was used to compare normal versus RPL conditions exclusively within the fetal aneuploid cell population.

### Machine learning model

We utilized devCellPy, a hierarchical machine learning framework built on XGBoost, to identify key features associated with the Norm and RPL conditions [42]. The dataset was stratified into training (90%) and testing (10%) sets to ensure robust performance evaluation. The classification model included two stages. In the first level, broad cell types were identified, and in the second level, immune cell types were distinguished based on the Norm and RPL conditions. We assessed the contribution of individual genes in the classification model by leveraging SHAP (Shapley Additive Explanations) analysis, which quantified the impact of each feature on the model’s predictions by attributing a specific importance value to each gene.

### Validation of prioritized RPL-associated genes using a transformer model of AI

To validate the SHAP-derived gene signatures for RPL immune signature identification, we employed scGPT, a transformer-based large language model specialized for single-cell RNA-seq data [46]. To determine the optimal feature set size, we evaluated model performance by varying the number of top SHAP-ranked genes (*n*) from both classification levels of devCellPy. Based on this optimization analysis, we selected the top 100 genes for each of the three immune cell types (T cells, granulocytes, and NK CD16 + cells). Using the pretrained scGPT_human model, we fine-tuned scGPT exclusively on these selected genes. We assessed both the zero-shot performance of the pre-trained model and the performance after fine-tuning. The fine-tuned models were subsequently used to classify cells into six categories: Tcell_Normal, Tcell_RPL, Granulocyte_Normal, Granulocyte_RPL, NK_CD16 ⁺ _Normal, and NK_CD16 ⁺ _RPL. This validation step ensured that the SHAP-ranked genes retained their predictive power across a different model architecture, supporting the applicability of the identified gene signatures to detect RPL-associated immune signatures.

### Drug repurposing

Drug repurposing analysis was performed using ASGARD [57]. As input, we used the signature gene list derived from the integration of patient and cell type information from the previous step. Therefore, patient identity and cell type were not separately used in this step. To ensure adequate coverage across drugs, we selected six tissues (lung, prostate, skin, breast, kidney, and large intestine) for which experimental data were available for at least 700 compounds. Drugs were selected if they were predicted to have significant effects (FDR < 0.05) in at least three of these tissues. Unless otherwise specified, all procedures followed the original ASGARD pipeline.

### Protein target and interaction data

Drug–target interactions were extracted from the DrugBank database [102]. Protein–protein interaction (PPI) data were obtained from the STRING database [103]. To ensure high-confidence interactions, we applied a confidence score threshold of 200 based on the STRING-provided scores. The optimal threshold was determined by varying the cutoff in increments of 100 and selecting the highest value that retained interactions involving all signature proteins and drug targets. To prioritize central hub proteins within the RPL network, we computed four distinct centrality measures using the NetworkX library (3.4.2) in Python [104]: degree centrality, betweenness centrality, eigenvector centrality, and random walk centrality. Proteins were ranked based on their average centrality scores to identify key regulatory nodes.

## Supporting information

S1 Fig Canonical marker gene expression supporting cell-type annotation. Dot plot showing the expression of three to five canonical marker genes for each major cell type and subtype identified in the decidual single-cell atlas. Dot size indicates the fraction of cells expressing each gene, and color intensity indicates the average scaled expression. The expected lineage-defining markers are enriched in their corresponding annotated populations, supporting the validity of the cell-type annotations.
(PDF)

S2 Fig Differential gene expression between Norm and RPL in euploid and aneuploid fetal cells. Volcano plots showing differentially expressed genes between Norm and RPL conditions, analyzed separately within the aneuploid (left) and euploid (right) fetal cell subsets. The x-axis indicates log2 fold change (RPL vs. Norm) and the y-axis indicates -log10(adjusted p-value). Genes with adjusted p < 0.05 and |log2 fold change| > 1 are highlighted (red, upregulated; blue, downregulated). Representative immune-activation and metabolic genes discussed in the main text are labeled.
(PDF)

S3 Fig Key gene expression changes across immune cell types between Norm and RPL. Heatmap showing the log2 fold changes (RPL vs. Norm) of representative inflammatory and tolerance-associated genes (CCL3, CCL4, CCL5, MIF, IGF2, CGA, SPP1) across decidual immune cell types. Color indicates the magnitude and direction of expression change. The coordinated upregulation of inflammatory chemokines and downregulation of tolerance-associated factors is most consistent in the dNK2 and dNK3 subsets.
(PDF)

S4 Fig Detailed performance of the classification model in the test sets. (A–B) The performance of the classification model was evaluated for each cell type in the test validation set, with weighted precision, recall, and F1 score shown for the 1st (A) and the 2nd (B) levels.
(PDF)

S5 Fig Detailed performance of our established classification model in the validation sets. (A–B) The performance of the classification model was evaluated for each cell type in Du’s dataset, with weighted precision, recall, and F1 score shown for the 1st (A) and 2nd (B) levels. (C) The weighted average precision, recall, and F1 score for each cell type at the 1st level evaluated on Vento’s dataset.
(PDF)

S1 Table Patient statistics. (XLSX)

S2 Table Cell count distribution. (XLSX)

S3 Table Differentially expressed genes between normal and RPL in aneuploid fetal cells. (XLSX)

S4 Table Differentially expressed genes between normal and RPL in euploid fetal cells. (XLSX)

S5 Table Differentially expressed genes in each cell type. (XLSX)

S6 Table SHAP analysis results for the first-level classification. (XLSX)

S7 Table SHAP analysis results for the second-level classification. (XLSX)

S8 Table Over-representation analysis results of RPL signatures. (XLSX)

S9 Table Tissue specific scores of drugs for reversing RPL-associated transcriptional changes. (XLSX)

S10 Table Centrality analysis result of T-cell RPL signature genes on the PPI network. (XLSX)

## References

[1] van Dijk MM, Kolte AM, Limpens J, Kirk E, Quenby S, van Wely M, et al. Recurrent pregnancy loss: diagnostic workup after two or three pregnancy losses? A systematic review of the literature and meta-analysis. Hum Reprod Update. 2020;26(3):356–67. doi: 10.1093/humupd/dmz048 32103270 PMC7161667

[2] Guleria I, Sayegh MH. Maternal acceptance of the fetus: true human tolerance. J Immunol. 2007;178(6):3345–51. doi: 10.4049/jimmunol.178.6.3345 17339426

[3] Alecsandru D, Klimczak AM, Garcia Velasco JA, Pirtea P, Franasiak JM. Immunologic causes and thrombophilia in recurrent pregnancy loss. Fertil Steril. 2021;115(3):561–6. doi: 10.1016/j.fertnstert.2021.01.017 33610320

[4] Practice Committee of the American Society for Reproductive Medicine. Evaluation and treatment of recurrent pregnancy loss: a committee opinion. Fertil Steril. 2012;98(5):1103–11. doi: 10.1016/j.fertnstert.2012.06.048 22835448

[5] Ford HB, Schust DJ. Recurrent pregnancy loss: etiology, diagnosis, and therapy. Rev Obstet Gynecol. 2009;2(2):76–83. 19609401 PMC2709325

[6] Deshmukh H, Way SS. Immunological Basis for Recurrent Fetal Loss and Pregnancy Complications. Annu Rev Pathol. 2019;14:185–210. doi: 10.1146/annurev-pathmechdis-012418-012743 30183507 PMC6566855

[7] Vento-Tormo R, Efremova M, Botting RA, Turco MY, Vento-Tormo M, Meyer KB, et al. Single-cell reconstruction of the early maternal-fetal interface in humans. Nature. 2018;563(7731):347–53. doi: 10.1038/s41586-018-0698-6 30429548 PMC7612850

[8] Suryawanshi H, Morozov P, Straus A, Sahasrabudhe N, Max KEA, Garzia A, et al. A single-cell survey of the human first-trimester placenta and decidua. Sci Adv. 2018;4(10):eaau4788. doi: 10.1126/sciadv.aau4788 PMC6209386 30402542

[9] Huang H, Liu C, Wagle MM, Yang P. Evaluation of deep learning-based feature selection for single-cell RNA sequencing data analysis. Genome Biol. 2023;24(1):259. doi: 10.1186/s13059-023-03100-x 37950331 PMC10638755

[10] Kong Y, Yu T. A Deep Neural Network Model using Random Forest to Extract Feature Representation for Gene Expression Data Classification. Sci Rep. 2018;8(1):16477. doi: 10.1038/s41598-018-34833-6 PMC6220289 30405137

[11] Choi SR, Lee M. Transformer Architecture and Attention Mechanisms in Genome Data Analysis: A Comprehensive Review. Biology (Basel). 2023;12(7):1033. doi: 10.3390/biology12071033 37508462 PMC10376273

[12] Traglia M, Croen LA, Jones KL, Heuer LS, Yolken R, Kharrazi M, et al. Cross-genetic determination of maternal and neonatal immune mediators during pregnancy. Genome Med. 2018;10(1):67. doi: 10.1186/s13073-018-0576-8 30134952 PMC6106874

[13] Paik H, Ko TL, Park M, Park JE, Bunis D, Sirota M, et al. Searching for influencers among placental immune cells in preeclampsia [Internet]. Cold Spring Harbor Laboratory; 2025 [cited 2025 May 23]. Available from:doi: 10.1101/2025.03.04.641363

[14] Heaton H, Talman AM, Knights A, Imaz M, Gaffney DJ, Durbin R, et al. Souporcell: robust clustering of single-cell RNA-seq data by genotype without reference genotypes. Nat Methods. 2020;17(6):615–20. doi: 10.1038/s41592-020-0820-1 32366989 PMC7617080

[15] McInnes L, Healy J, Melville J. UMAP: Uniform Manifold Approximation and Projection for Dimension Reduction [Internet]. arXiv; 2020 [cited 2025 Feb 24]. Available from: http://arxiv.org/abs/1802.03426

[16] Stonier SW, Schluns KS. Trans-presentation: a novel mechanism regulating IL-15 delivery and responses. Immunol Lett. 2010;127(2):85–92. doi: 10.1016/j.imlet.2009.09.009 19818367 PMC2808451

[17] Xu T, Gao S, Liu J, Huang Y, Chen K, Zhang X. MMP9 and IGFBP1 Regulate Tumor Immune and Drive Tumor Progression in Clear Cell Renal Cell Carcinoma. J Cancer. 2021;12(8):2243–57. doi: 10.7150/jca.48664 33758602 PMC7974879

[18] Cai J, Chen Q, Cui Y, Dong J, Chen M, Wu P, et al. Immune heterogeneity and clinicopathologic characterization of IGFBP2 in 2447 glioma samples. Oncoimmunology. 2018;7(5):e1426516. doi: 10.1080/2162402X.2018.1426516 29721393 PMC5927515

[19] Liu J, Yin J, Wang Y, Cai L, Geng R, Du M, et al. A comprehensive prognostic and immune analysis of enhancer RNA identifies IGFBP7-AS1 as a novel prognostic biomarker in Uterine Corpus Endometrial Carcinoma. Biol Proced Online. 2022;24(1):9. doi: 10.1186/s12575-022-00172-0 35836132 PMC9284715

[20] Jain R, Chen Y, Kanno Y, Joyce-Shaikh B, Vahedi G, Hirahara K, et al. Interleukin-23-Induced Transcription Factor Blimp-1 Promotes Pathogenicity of T Helper 17 Cells. Immunity. 2016;44(1):131–42. doi: 10.1016/j.immuni.2015.11.009 26750311 PMC11608061

[21] Fernandez IZ, Baxter RM, Garcia-Perez JE, Vendrame E, Ranganath T, Kong DS, et al. A novel human IL2RB mutation results in T and NK cell-driven immune dysregulation. J Exp Med. 2019;216(6):1255–67. doi: 10.1084/jem.20182015 31040184 PMC6547857

[22] Shouse AN, LaPorte KM, Malek TR. Interleukin-2 signaling in the regulation of T cell biology in autoimmunity and cancer. Immunity. 2024;57(3):414–28. doi: 10.1016/j.immuni.2024.02.001 38479359 PMC11126276

[23] Pivniouk VI, Geha RS. The role of SLP-76 and LAT in lymphocyte development. Curr Opin Immunol. 2000;12(2):173–8. doi: 10.1016/s0952-7915(99)00068-0 10712938

[24] Tjong WY, Lin HH. The RGD motif is involved in CD97/ADGRE5-promoted cell adhesion and viability of HT1080 cells. Sci Rep. 2019;9(1):1517. doi: 10.1038/s41598-018-38045-w 30728423 PMC6365523

[25] Stepanek O, Kalina T, Draber P, Skopcova T, Svojgr K, Angelisova P, et al. Regulation of Src family kinases involved in T cell receptor signaling by protein-tyrosine phosphatase CD148. J Biol Chem. 2011;286(25):22101–12. doi: 10.1074/jbc.M110.196733 21543337 PMC3121354

[26] Szczypka M. Role of Phosphodiesterase 7 (PDE7) in T Cell Activity. Effects of Selective PDE7 Inhibitors and Dual PDE4/7 Inhibitors on T Cell Functions. Int J Mol Sci. 2020;21(17):6118. doi: 10.3390/ijms21176118 32854348 PMC7504236

[27] Johansen KH, Golec DP, Okkenhaug K, Schwartzberg PL. Mind the GAP: RASA2 and RASA3 GTPase-activating proteins as gatekeepers of T cell activation and adhesion. Trends Immunol. 2023;44(11):917–31. doi: 10.1016/j.it.2023.09.002 37858490 PMC10621891

[28] Chen ZX, Pervaiz S. Involvement of cytochrome c oxidase subunits Va and Vb in the regulation of cancer cell metabolism by Bcl-2. Cell Death Differ. 2010;17(3):408–20. doi: 10.1038/cdd.2009.132 19834492

[29] Knapp-Wilson A, Pereira GC, Buzzard E, Ford HC, Richardson A, Corey RA, et al. Maintenance of complex I and its supercomplexes by NDUF-11 is essential for mitochondrial structure, function and health. J Cell Sci. 2021;134(13):jcs258399. doi: 10.1242/jcs.258399 34106255 PMC8277142

[30] Tomasello MF, Guarino F, Reina S, Messina A, De Pinto V. The voltage-dependent anion selective channel 1 (VDAC1) topography in the mitochondrial outer membrane as detected in intact cell. PLoS One. 2013;8(12):e81522. doi: 10.1371/journal.pone.0081522 24324700 PMC3855671

[31] Wonsey DR, Zeller KI, Dang CV. The c-Myc target gene PRDX3 is required for mitochondrial homeostasis and neoplastic transformation. Proc Natl Acad Sci U S A. 2002;99(10):6649–54. doi: 10.1073/pnas.102523299 12011429 PMC124457

[32] Kocaturk NM, Peker N, Eberhart K, Akkoc Y, Deveci G, Dengjel J, et al. Novel protein complexes containing autophagy and UPS components regulate proteasome-dependent PARK2 recruitment onto mitochondria and PARK2-PARK6 activity during mitophagy. Cell Death Dis. 2022;13(11):947. doi: 10.1038/s41419-022-05339-x 36357363 PMC9649694

[33] Sudhan DR, Siemann DW. Cathepsin L targeting in cancer treatment. Pharmacol Ther. 2015;155:105–16. doi: 10.1016/j.pharmthera.2015.08.007 26299995 PMC4624022

[34] Khatri P, Roedder S, Kimura N, De Vusser K, Morgan AA, Gong Y, et al. A common rejection module (CRM) for acute rejection across multiple organs identifies novel therapeutics for organ transplantation. J Exp Med. 2013;210(11):2205–21. doi: 10.1084/jem.20122709 24127489 PMC3804941

[35] Wei X-W, Zhang Y-C, Wu F, Tian F-J, Lin Y. The role of extravillous trophoblasts and uterine NK cells in vascular remodeling during pregnancy. Front Immunol. 2022;13:951482. doi: 10.3389/fimmu.2022.951482 37408837 PMC10319396

[36] Huhn O, Ivarsson MA, Gardner L, Hollinshead M, Stinchcombe JC, Chen P, et al. Distinctive phenotypes and functions of innate lymphoid cells in human decidua during early pregnancy. Nat Commun. 2020;11(1):381. doi: 10.1038/s41467-019-14123-z 31959757 PMC6971012

[37] Nancy P, Tagliani E, Tay CS, Asp P, Levy DE, Erlebacher A. Chemokine gene silencing in decidual stromal cells limits T cell access to the maternal-fetal interface. Science. 2012;336(6086):1317–21. doi: 10.1126/science.1220030 22679098 PMC3727649

[38] Ietta F, Todros T, Ticconi C, Piccoli E, Zicari A, Piccione E, et al. Macrophage migration inhibitory factor in human pregnancy and labor. Am J Reprod Immunol N Y N 1989. 2002;48(6):404–9. doi: 10.1034/j.1600-0897.2002.01152.x 12607777

[39] Harris LK, Crocker IP, Baker PN, Aplin JD, Westwood M. IGF2 actions on trophoblast in human placenta are regulated by the insulin-like growth factor 2 receptor, which can function as both a signaling and clearance receptor. Biol Reprod. 2011;84(3):440–6. doi: 10.1095/biolreprod.110.088195 20980691 PMC3043127

[40] Tsampalas M, Gridelet V, Berndt S, Foidart J-M, Geenen V, Perrier d’Hauterive S. Human chorionic gonadotropin: a hormone with immunological and angiogenic properties. J Reprod Immunol. 2010;85(1):93–8. doi: 10.1016/j.jri.2009.11.008 20227765

[41] Yi X, Luo L, Zhu Y, Deng H, Liao H, Shen Y, et al. SPP1 facilitates cell migration and invasion by targeting COL11A1 in lung adenocarcinoma. Cancer Cell Int. 2022;22(1):324. doi: 10.1186/s12935-022-02749-x 36266702 PMC9583566

[42] Galdos FX, Xu S, Goodyer WR, Duan L, Huang YV, Lee S, et al. devCellPy is a machine learning-enabled pipeline for automated annotation of complex multilayered single-cell transcriptomic data. Nat Commun. 2022;13(1):5271. doi: 10.1038/s41467-022-33045-x 36071107 PMC9452519

[43] Du L, Deng W, Zeng S, Xu P, Huang L, Liang Y, et al. Single-cell transcriptome analysis reveals defective decidua stromal niche attributes to recurrent spontaneous abortion. Cell Prolif. 2021;54(11):e13125. doi: 10.1111/cpr.13125 34546587 PMC8560595

[44] Lundberg SM, Lee SI. A unified approach to interpreting model predictions. In: Proceedings of the 31st International Conference on Neural Information Processing Systems. Red Hook, NY, USA: Curran Associates Inc.; 2017. p. 4768–77. (NIPS’17).

[45] Abou-Kheir W, Eid A, El-Merahbi R, Assaf R, Daoud G. A Unique Expression of Keratin 14 in a Subset of Trophoblast Cells. Milstone DS, editor. PLOS ONE. 2015;10(10):e0139939. doi: 10.1371/journal.pone.0139939 PMC4592186 26430881

[46] Cui H, Wang C, Maan H, Pang K, Luo F, Duan N, et al. scGPT: toward building a foundation model for single-cell multi-omics using generative AI. Nat Methods. 2024;21(8):1470–80. doi: 10.1038/s41592-024-02201-0 38409223

[47] Araki K, Morita M, Bederman AG, Konieczny BT, Kissick HT, Sonenberg N, et al. Translation is actively regulated during the differentiation of CD8+ effector T cells. Nat Immunol. 2017;18(9):1046–57. doi: 10.1038/ni.3795 28714979 PMC5937989

[48] Wolf T, Jin W, Zoppi G, Vogel IA, Akhmedov M, Bleck CKE, et al. Dynamics in protein translation sustaining T cell preparedness. Nat Immunol. 2020;21(8):927–37. doi: 10.1038/s41590-020-0714-5 32632289 PMC7610365

[49] Marchingo JM, Cantrell DA. Protein synthesis, degradation, and energy metabolism in T cell immunity. Cell Mol Immunol. 2022;19(3):303–15. doi: 10.1038/s41423-021-00792-8 34983947 PMC8891282

[50] Hug N, Longman D, Cáceres JF. Mechanism and regulation of the nonsense-mediated decay pathway. Nucleic Acids Res. 2016;44(4):1483–95. doi: 10.1093/nar/gkw010 26773057 PMC4770240

[51] Akiyama T, Suzuki T, Yamamoto T. RNA decay machinery safeguards immune cell development and immunological responses. Trends Immunol. 2021;42(5):447–60. doi: 10.1016/j.it.2021.03.008 33858774

[52] Li P, Shi Y, Shuai H, Cai Y, Lu W, Wang G, et al. Alterted SLIT2/ROBO1 signalling is linked to impaired placentation of missed and threatened miscarriage in early pregnancy. Histopathology. 2017;71(4):543–52. doi: 10.1111/his.13250 28485101

[53] Davalieva K, Terzikj M, Bozhinovski G, Kiprijanovska S, Kubelka-Sabit K, Plaseska-Karanfilska D. Comparative proteomics analysis of decidua reveals altered RNA processing and impaired ribosome function in recurrent pregnancy loss. Placenta. 2024;154:28–37. doi: 10.1016/j.placenta.2024.06.005 38870839

[54] Racicot K, Mor G. Risks associated with viral infections during pregnancy. J Clin Invest. 2017;127(5):1591–9. doi: 10.1172/JCI87490 28459427 PMC5409792

[55] Nigro G, Mazzocco M, Mattia E, Di Renzo GC, Carta G, Anceschi MM. Role of the infections in recurrent spontaneous abortion. J Matern Fetal Neonatal Med. 2011;24(8):983–9. doi: 10.3109/14767058.2010.547963 21261443

[56] Kedia-Mehta N, Finlay DK. Competition for nutrients and its role in controlling immune responses. Nat Commun. 2019;10(1):2123. doi: 10.1038/s41467-019-10015-4 31073180 PMC6509329

[57] He B, Xiao Y, Liang H, Huang Q, Du Y, Li Y, et al. ASGARD is A Single-cell Guided Pipeline to Aid Repurposing of Drugs. Nat Commun. 2023;14(1):993. doi: 10.1038/s41467-023-36637-3 36813801 PMC9945835

[58] Kemp MW, Newnham JP, Challis JG, Jobe AH, Stock SJ. The clinical use of corticosteroids in pregnancy. Hum Reprod Update. 2016;22(2):240–59. doi: 10.1093/humupd/dmv047 26590298

[59] Dan S, Wei W, Yichao S, Hongbo C, Shenmin Y, Jiaxiong W, et al. Effect of Prednisolone Administration on Patients with Unexplained Recurrent Miscarriage and in Routine Intracytoplasmic Sperm Injection: A Meta-Analysis. Am J Reprod Immunol N Y N 1989. 2015;74(1):89–97. doi: 10.1111/aji.12373 25753479

[60] D’Ippolito S, Gavi F, Granieri C, De Waure C, Giuliano S, Cosentino F, et al. Efficacy of Corticosteroids in Patients With Recurrent Pregnancy Loss: A Systematic Review and Meta-Analysis. Am J Reprod Immunol N Y N 1989. 2025;93(1):e70037. doi: 10.1111/aji.70037 39777851

[61] Royster GD, Harris JC, Nelson A, Castro Y, Weitzel RP, Tisdale J, et al. Rapamycin Corrects T Regulatory Cell Depletion and Improves Embryo Implantation and Live Birth Rates in a Murine Model. Reprod Sci Thousand Oaks Calif. 2019;26(12):1545–56. doi: 10.1177/1933719119828110 30782087 PMC6949958

[62] Lu H, Yang H-L, Zhou W-J, Lai Z-Z, Qiu X-M, Fu Q, et al. Rapamycin prevents spontaneous abortion by triggering decidual stromal cell autophagy-mediated NK cell residence. Autophagy. 2021;17(9):2511–27. doi: 10.1080/15548627.2020.1833515 33030400 PMC8496707

[63] Ahmadi M, Abdolmohamadi-Vahid S, Ghaebi M, Dolati S, Abbaspour-Aghdam S, Danaii S, et al. Sirolimus as a new drug to treat RIF patients with elevated Th17/Treg ratio: A double-blind, phase II randomized clinical trial. Int Immunopharmacol. 2019;74:105730. doi: 10.1016/j.intimp.2019.105730 31299610

[64] Morin F, Kavian N, Nicco C, Cerles O, Chéreau C, Batteux F. Niclosamide Prevents Systemic Sclerosis in a Reactive Oxygen Species-Induced Mouse Model. J Immunol. 2016;197(8):3018–28. doi: 10.4049/jimmunol.1502482 27613696

[65] Jang SG, Lee J, Hong SM, Song YS, Kim MJ, Kwok SK, et al. Niclosamide suppresses the expansion of follicular helper T cells and alleviates disease severity in two murine models of lupus via STAT3. J Transl Med. 2021;19(1):86. doi: 10.1186/s12967-021-02760-2 33632240 PMC7908700

[66] Takada K, Kashiwagi S, Asano Y, Goto W, Takahashi K, Fujita H, et al. Verification of the effects of calcium channel blockers on the immune microenvironment of breast cancer. BMC Cancer. 2019;19(1):615. doi: 10.1186/s12885-019-5828-5 31234828 PMC6591916

[67] Veytia-Bucheli JI, Alvarado-Velázquez DA, Possani LD, González-Amaro R, Rosenstein Y. The Ca2+ Channel Blocker Verapamil Inhibits the In Vitro Activation and Function of T Lymphocytes: A 2022 Reappraisal. Pharmaceutics. 2022;14(7):1478. doi: 10.3390/pharmaceutics14071478 35890372 PMC9324055

[68] Barabási A-L, Oltvai ZN. Network biology: understanding the cell’s functional organization. Nat Rev Genet. 2004;5(2):101–13. doi: 10.1038/nrg1272 14735121

[69] Ashtiani M, Salehzadeh-Yazdi A, Razaghi-Moghadam Z, Hennig H, Wolkenhauer O, Mirzaie M, et al. A systematic survey of centrality measures for protein-protein interaction networks. BMC Syst Biol. 2018;12(1):80. doi: 10.1186/s12918-018-0598-2 30064421 PMC6069823

[70] Pozzobon T, Goldoni G, Viola A, Molon B. CXCR4 signaling in health and disease. Immunol Lett. 2016;177:6–15. doi: 10.1016/j.imlet.2016.06.006 27363619

[71] Cargnin S, Agnusdei F, Shin JI, Terrazzino S. Maternal ApoE genotype and risk of recurrent pregnancy loss: An updated systematic review and meta-analysis. J Gene Med. 2023;25(3):e3467. doi: 10.1002/jgm.3467 36479790

[72] Turienzo A, Lledó B, Ortiz JA, Morales R, Sanz J, Llácer J, et al. Prevalence of candidate single nucleotide polymorphisms on p53, IL-11, IL-10, VEGF and APOE in patients with repeated implantation failure (RIF) and pregnancy loss (RPL). Hum Fertil (Camb). 2020;23(2):117–22. doi: 10.1080/14647273.2018.1524935 30332901

[73] Li P, Spolski R, Liao W, Wang L, Murphy TL, Murphy KM, et al. BATF-JUN is critical for IRF4-mediated transcription in T cells. Nature. 2012;490(7421):543–6. doi: 10.1038/nature11530 22992523 PMC3537508

[74] Lynn RC, Weber EW, Sotillo E, Gennert D, Xu P, Good Z, et al. c-Jun overexpression in CAR T cells induces exhaustion resistance. Nature. 2019;576(7786):293–300. doi: 10.1038/s41586-019-1805-z 31802004 PMC6944329

[75] Millar DG, Garza KM, Odermatt B, Elford AR, Ono N, Li Z, et al. Hsp70 promotes antigen-presenting cell function and converts T-cell tolerance to autoimmunity in vivo. Nat Med. 2003;9(12):1469–76. doi: 10.1038/nm962 14625545

[76] Colgan J, Asmal M, Neagu M, Yu B, Schneidkraut J, Lee Y, et al. Cyclophilin A regulates TCR signal strength in CD4+ T cells via a proline-directed conformational switch in Itk. Immunity. 2004;21(2):189–201. doi: 10.1016/j.immuni.2004.07.005 15308100

[77] Chen C-T, Shan C-X, Ran J, Yin L-M, Li H-Y, Wang Y, et al. Cyclophilin A Plays Potential Roles in a Rat Model of Asthma and Suppression of Immune Response. J Asthma Allergy. 2021;14:471–80. doi: 10.2147/JAA.S308938 33994799 PMC8114824

[78] Musaelyan A, Lapin S, Nazarov V, Tkachenko O, Gilburd B, Mazing A, et al. Vimentin as antigenic target in autoimmunity: A comprehensive review. Autoimmun Rev. 2018;17(9):926–34. doi: 10.1016/j.autrev.2018.04.004 30009963

[79] McDonald-Hyman C, Muller JT, Loschi M, Thangavelu G, Saha A, Kumari S, et al. The vimentin intermediate filament network restrains regulatory T cell suppression of graft-versus-host disease. J Clin Invest. 2018;128(10):4604–21. doi: 10.1172/JCI95713 30106752 PMC6159973

[80] Mousavi A. CXCL12/CXCR4 signal transduction in diseases and its molecular approaches in targeted-therapy. Immunol Lett. 2020;217:91–115. doi: 10.1016/j.imlet.2019.11.007 31747563

[81] Lyu F, Burzynski C, Fang YY, Tal A, Chen AY, Kisa J, et al. Maternal CXCR4 deletion results in placental defects and pregnancy loss mediated by immune dysregulation. JCI Insight. 2023;8(21):e172216. doi: 10.1172/jci.insight.172216 37815869 PMC10721256

[82] Tao Y, Li Y, Zhang D, Xu L, Chen J, Sang Y, et al. Decidual CXCR4+CD56brightNK cells as a novel NK subset in maternal–foetal immune tolerance to alleviate early pregnancy failure. Clin Transl Med. 2021;11(10):e540. doi: 10.1002/ctm2.540 34709764 PMC8516340

[83] Ramonell KM, Zhang W, Hadley A, Chen C-W, Fay KT, Lyons JD, et al. CXCR4 blockade decreases CD4+ T cell exhaustion and improves survival in a murine model of polymicrobial sepsis. PLoS One. 2017;12(12):e0188882. doi: 10.1371/journal.pone.0188882 29232699 PMC5726761

[84] Guo W, Huai Q, Zhou B, Guo L, Sun L, Xue X, et al. Comprehensive analysis of the immunological implication and prognostic value of CXCR4 in non-small cell lung cancer. Cancer Immunol Immunother. 2023;72(4):1029–45. doi: 10.1007/s00262-022-03298-y 36308553 PMC10025233

[85] Mognol GP, González-Avalos E, Ghosh S, Spreafico R, Gudlur A, Rao A, et al. Targeting the NFAT:AP-1 transcriptional complex on DNA with a small-molecule inhibitor. Proc Natl Acad Sci U S A. 2019;116(20):9959–68. doi: 10.1073/pnas.1820604116 31019078 PMC6525529

[86] Shaulian E, Karin M. AP-1 as a regulator of cell life and death. Nat Cell Biol. 2002;4(5):E131–6. doi: 10.1038/ncb0502-e131 11988758

[87] Yukawa M, Jagannathan S, Vallabh S, Kartashov AV, Chen X, Weirauch MT, et al. AP-1 activity induced by co-stimulation is required for chromatin opening during T cell activation. J Exp Med. 2019;217(1):e20182009. doi: 10.1084/jem.20182009 31653690 PMC7037242

[88] Torgerson TR, Colosia AD, Donahue JP, Lin YZ, Hawiger J. Regulation of NF-κB, AP-1, NFAT, and STAT1 Nuclear Import in T Lymphocytes by Noninvasive Delivery of Peptide Carrying the Nuclear Localization Sequence of NF-κB p501. J Immunol. 1998;161(11):6084–92. doi: 10.4049/jimmunol.161.11.6084 9834092

[89] Peng B, Zhu H, Ma L, Wang YL, Klausen C, Leung PCK. AP-1 Transcription Factors c-FOS and c-JUN Mediate GnRH-Induced Cadherin-11 Expression and Trophoblast Cell Invasion. Endocrinology. 2015;156(6):2269–77. doi: 10.1210/en.2014-1871 25794160

[90] Zuo S, Li C, Sun X, Deng B, Zhang Y, Han Y, et al. C-JUN overexpressing CAR-T cells in acute myeloid leukemia: preclinical characterization and phase I trial. Nat Commun. 2024;15(1):6155. doi: 10.1038/s41467-024-50485-9 39039086 PMC11263573

[91] Yang F, Zheng Q, Jin L. Dynamic Function and Composition Changes of Immune Cells During Normal and Pathological Pregnancy at the Maternal-Fetal Interface. Front Immunol. 2019;10:2317. doi: 10.3389/fimmu.2019.02317 31681264 PMC6813251

[92] Young MD, Behjati S. SoupX removes ambient RNA contamination from droplet-based single-cell RNA sequencing data. GigaScience. 2020;9(12):giaa151. doi: 10.1093/gigascience/giaa151 PMC7763177 33367645

[93] Wolock SL, Lopez R, Klein AM. Scrublet: Computational Identification of Cell Doublets in Single-Cell Transcriptomic Data. Cell Syst. 2019;8(4):281–291.e9. doi: 10.1016/j.cels.2018.11.005 30954476 PMC6625319

[94] Korsunsky I, Millard N, Fan J, Slowikowski K, Zhang F, Wei K, et al. Fast, sensitive and accurate integration of single-cell data with Harmony. Nat Methods. 2019;16(12):1289–96. doi: 10.1038/s41592-019-0619-0 31740819 PMC6884693

[95] Polański K, Young MD, Miao Z, Meyer KB, Teichmann SA, Park J-E. BBKNN: fast batch alignment of single cell transcriptomes. Bioinformatics. 2020;36(3):964–5. doi: 10.1093/bioinformatics/btz625 31400197 PMC9883685

[96] Domínguez Conde C, Xu C, Jarvis LB, Rainbow DB, Wells SB, Gomes T, et al. Cross-tissue immune cell analysis reveals tissue-specific features in humans. Science. 2022;376(6594):eabl5197. doi: 10.1126/science.abl5197 PMC7612735 35549406

[97] Starostik MR, Sosina OA, McCoy RC. Single-cell analysis of human embryos reveals diverse patterns of aneuploidy and mosaicism. Genome Res. 2020;30(6):814–25. doi: 10.1101/gr.262774.120 32641298 PMC7370883

[98] Griffiths JA, Scialdone A, Marioni JC. Mosaic autosomal aneuploidies are detectable from single-cell RNAseq data. BMC Genomics. 2017;18(1):904. doi: 10.1186/s12864-017-4253-x 29178830 PMC5702132

[99] Li H, Handsaker B, Wysoker A, Fennell T, Ruan J, Homer N, et al. The Sequence Alignment/Map format and SAMtools. Bioinformatics. 2009;25(16):2078–9. doi: 10.1093/bioinformatics/btp352 PMC2723002 19505943

[100] Van der Auwera GA, Carneiro MO, Hartl C, Poplin R, Del Angel G, Levy-Moonshine A, et al. From FastQ data to high confidence variant calls: the Genome Analysis Toolkit best practices pipeline. Curr Protoc Bioinformatics. 2013;43(1110):11.10.1–11.10.33. doi: 10.1002/0471250953.bi1110s43 25431634 PMC4243306

[101] Love MI, Huber W, Anders S. Moderated estimation of fold change and dispersion for RNA-seq data with DESeq2. Genome Biol. 2014;15(12):550. doi: 10.1186/s13059-014-0550-8 25516281 PMC4302049

[102] Knox C, Wilson M, Klinger CM, Franklin M, Oler E, Wilson A, et al. DrugBank 6.0: the DrugBank Knowledgebase for 2024. Nucleic Acids Res. 2024;52(D1):D1265–75. doi: 10.1093/nar/gkad976 37953279 PMC10767804

[103] Szklarczyk D, Kirsch R, Koutrouli M, Nastou K, Mehryary F, Hachilif R, et al. The STRING database in 2023: protein-protein association networks and functional enrichment analyses for any sequenced genome of interest. Nucleic Acids Res. 2023;51(D1):D638–46. doi: 10.1093/nar/gkac1000 36370105 PMC9825434

[104] Hagberg AA, Schult DA, Swart PJ. Exploring Network Structure, Dynamics, and Function using Network. In: Proceedings of the Python in Science Conference [Internet]. Pasadena, California: SciPy; 2008 [cited 2025 Jul 24]. p. 11–5. Available from: https://doi.curvenote.com/10.25080/TCWV9851

