# Supplementary material for: Machine learning–driven decoding of maternal immune signatures in repeated pregnancy loss
Source: PLoS Comput Biol. 2026 Sep 17;22(9):e1014806. doi: 10.1371/journal.pcbi.1014806 (PMC13619008; doi:10.1371/journal.pcbi.1014806)

Canonical Marker Gene Expression by Cell Type (Grouped)
(Vento-Tormo et al., 2018)

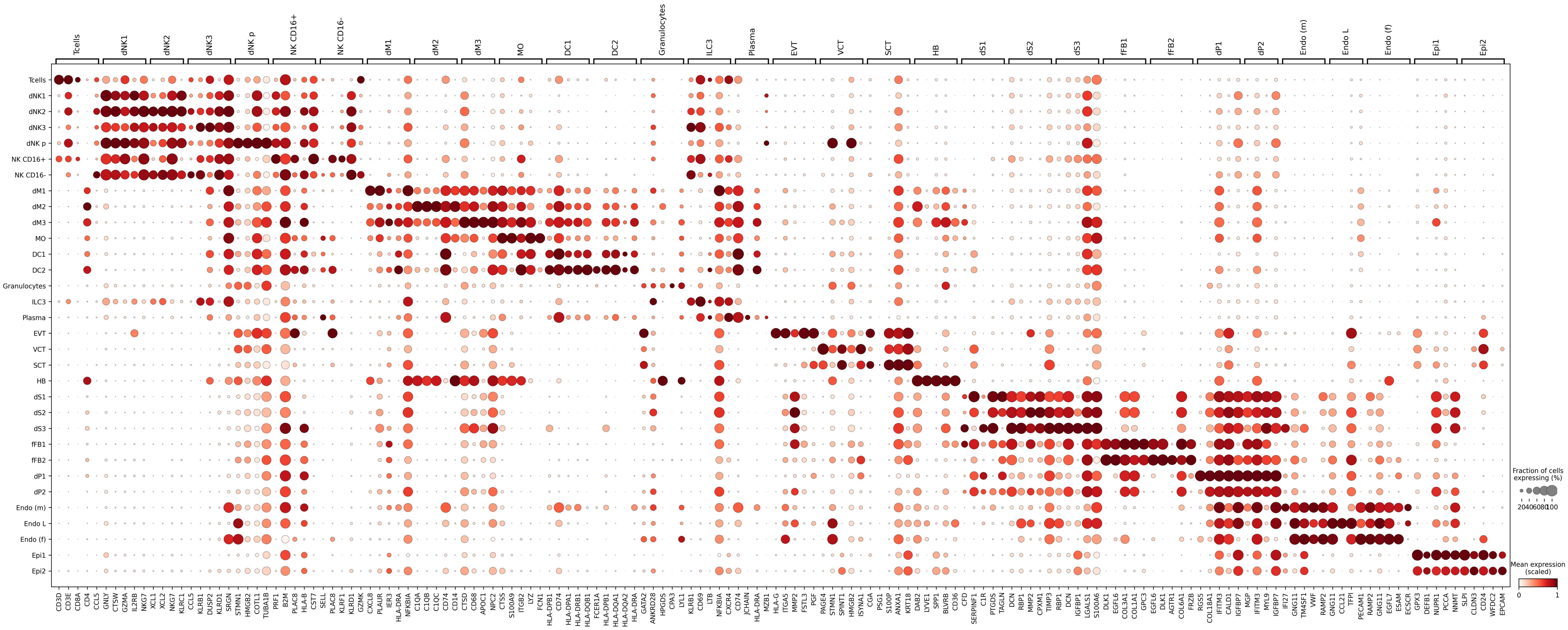

Supplement: Supplementary file 1 [file pcbi.1014806.s001.pdf]
