# Supplementary material for: Machine learning–driven decoding of maternal immune signatures in repeated pregnancy loss
Source: PLoS Comput Biol. 2026 Sep 17;22(9):e1014806. doi: 10.1371/journal.pcbi.1014806 (PMC13619008; doi:10.1371/journal.pcbi.1014806)
Supplement: Supplementary file 2 [file pcbi.1014806.s002.pdf]

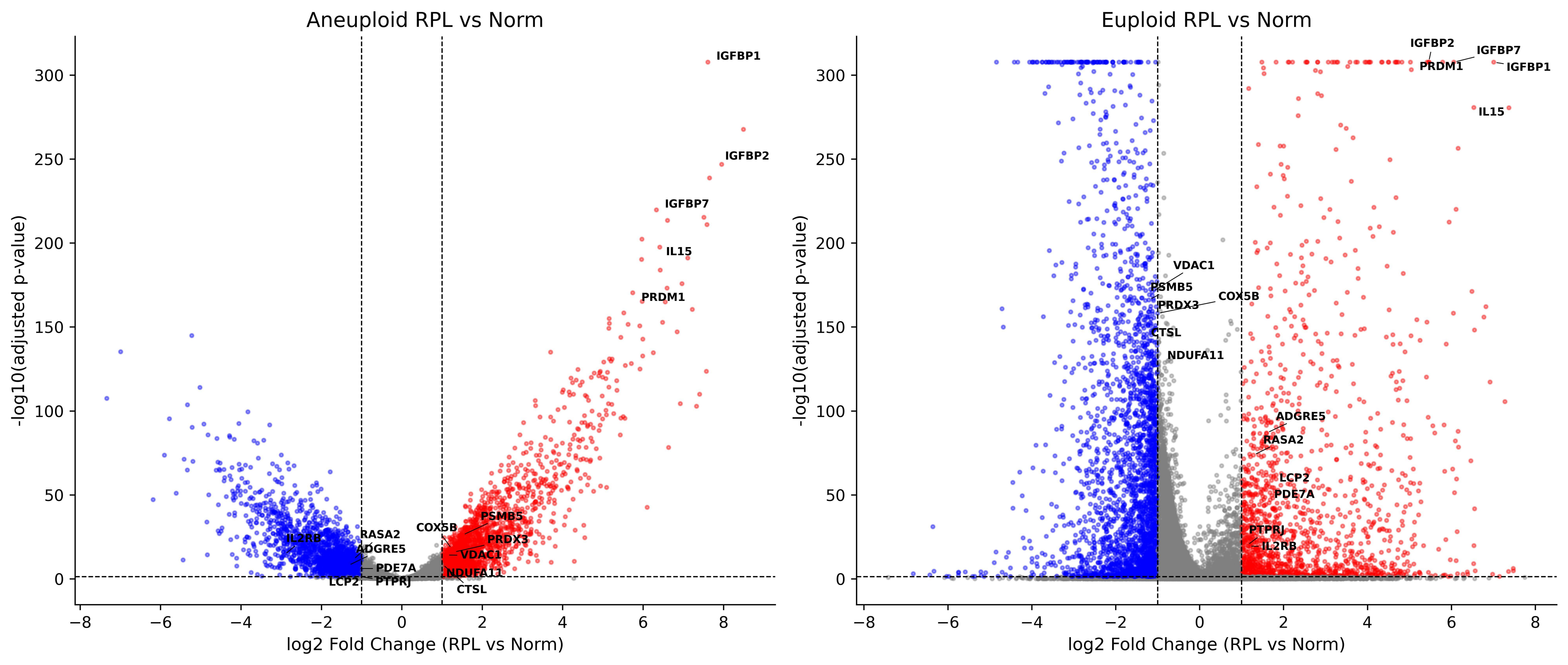
Aneuploid RPL vs Norm
IGFBP1
IGFBP2
IGFBP7
IL15
PRDM1
PSMB5
COX5B
PRDX3
VDAC1
NDUFA11
CTSL
IL2RB
RASA2
ADGRE5
PDE7A
LCP2
PTPRJ
-log10(adjusted p-value)
log2 Fold Change (RPL vs Norm)
Euploid RPL vs Norm
IGFBP2
IGFBP7
PRDM1
IGFBP1
IL15
VDAC1
PSMB5
COX5B
PRDX3
CTSL
NDUFA11
ADGRE5
RASA2
LCP2
PDE7A
PTPRJ
IL2RB
-log10(adjusted p-value)
log2 Fold Change (RPL vs Norm)
