# Supplementary material for: Machine learning–driven decoding of maternal immune signatures in repeated pregnancy loss
Source: PLoS Comput Biol. 2026 Sep 17;22(9):e1014806. doi: 10.1371/journal.pcbi.1014806 (PMC13619008; doi:10.1371/journal.pcbi.1014806)
Supplement: Supplementary file 3 [file pcbi.1014806.s003.pdf]

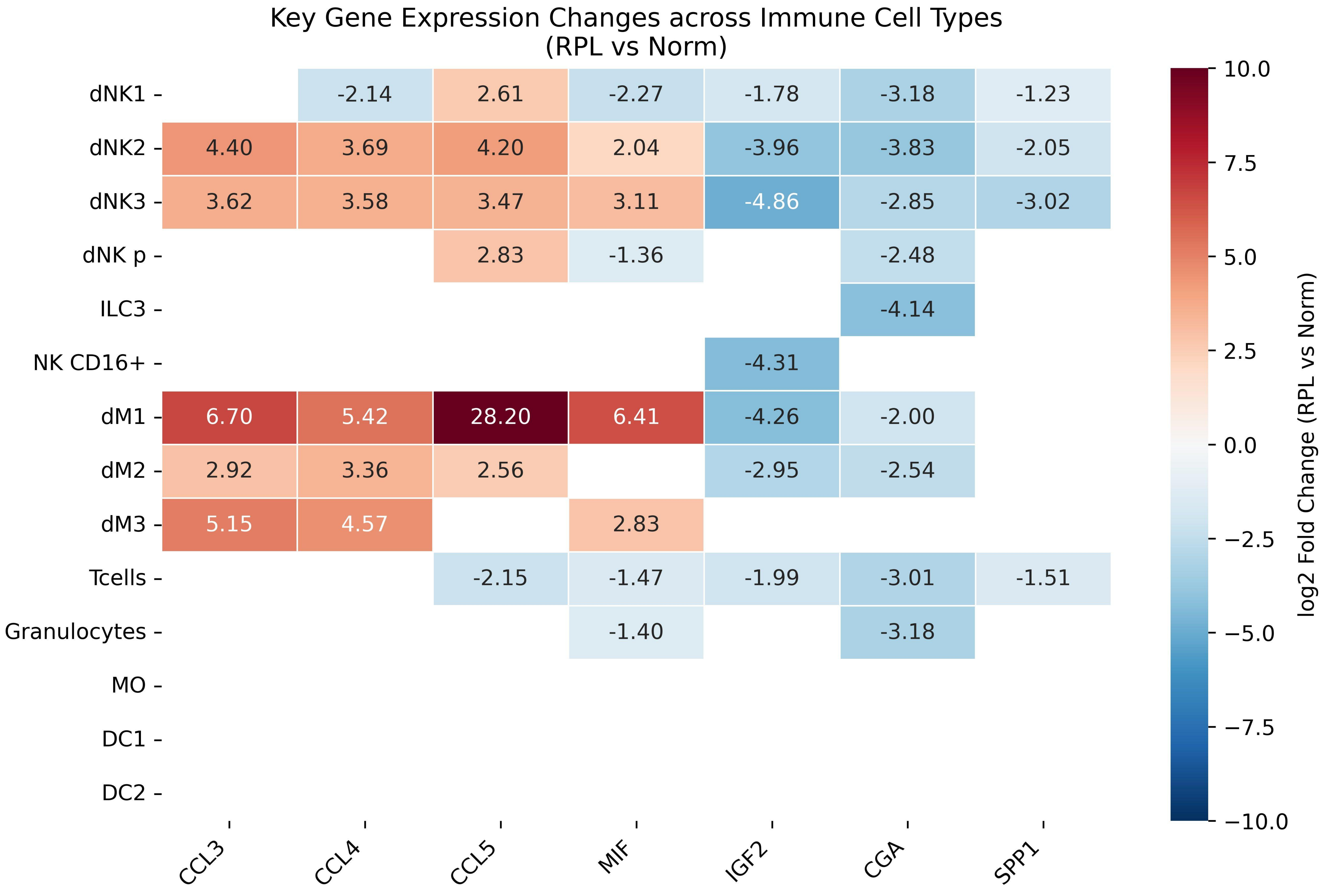
Key Gene Expression Changes across Immune Cell Types
(RPL vs Norm)
dNK1
dNK2
dNK3
dNK p
ILC3
NK CD16+
dM1
dM2
dM3
Tcells
Granulocytes
MO
DC1
DC2
CCL3
CCL4
CCL5
MIF
IGF2
CGA
SPP1
-2.14 2.61 -2.27 -1.78 -3.18 -1.23
4.40 3.69 4.20 2.04 -3.96 -3.83 -2.05
3.62 3.58 3.47 3.11 -4.86 -2.85 -3.02
2.83 -1.36 -2.48
-4.14
-4.31
6.70 5.42 28.20 6.41 -4.26 -2.00
2.92 3.36 2.56 -2.95 -2.54
5.15 4.57 2.83
-2.15 -1.47 -1.99 -3.01 -1.51
-1.40 -3.18
log2 Fold Change (RPL vs Norm)
10.0
7.5
5.0
2.5
0.0
−2.5
−5.0
−7.5
−10.0
