# Supplementary material for: Machine learning–driven decoding of maternal immune signatures in repeated pregnancy loss
Source: PLoS Comput Biol. 2026 Sep 17;22(9):e1014806. doi: 10.1371/journal.pcbi.1014806 (PMC13619008; doi:10.1371/journal.pcbi.1014806)

A.

<Test validation set>

1st level (32 labels)

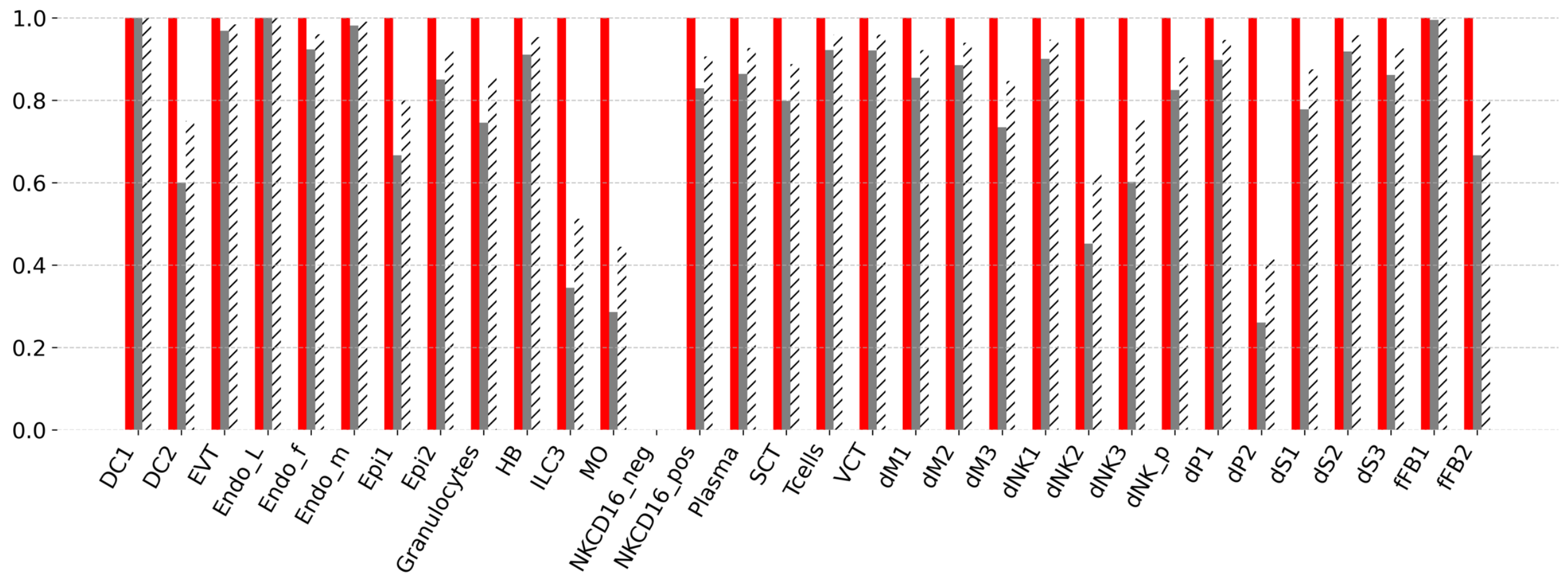

B.

<Test validation set>

2nd level (34 labels)

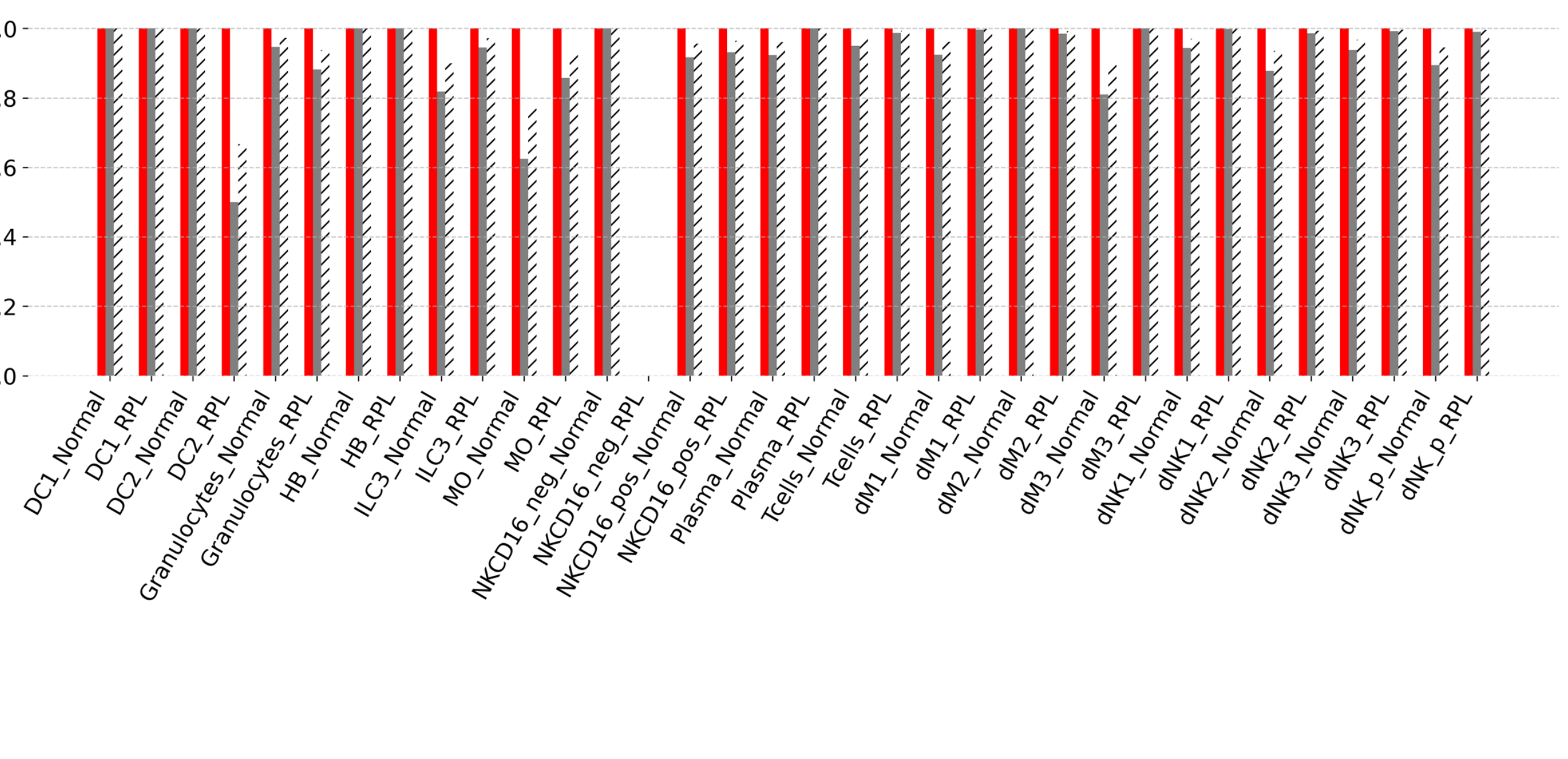

Supplement: Supplementary file 4 [file pcbi.1014806.s004.pdf]
