# Supplementary material for: Machine learning–driven decoding of maternal immune signatures in repeated pregnancy loss
Source: PLoS Comput Biol. 2026 Sep 17;22(9):e1014806. doi: 10.1371/journal.pcbi.1014806 (PMC13619008; doi:10.1371/journal.pcbi.1014806)

A.

<Du's dataset>

1st level

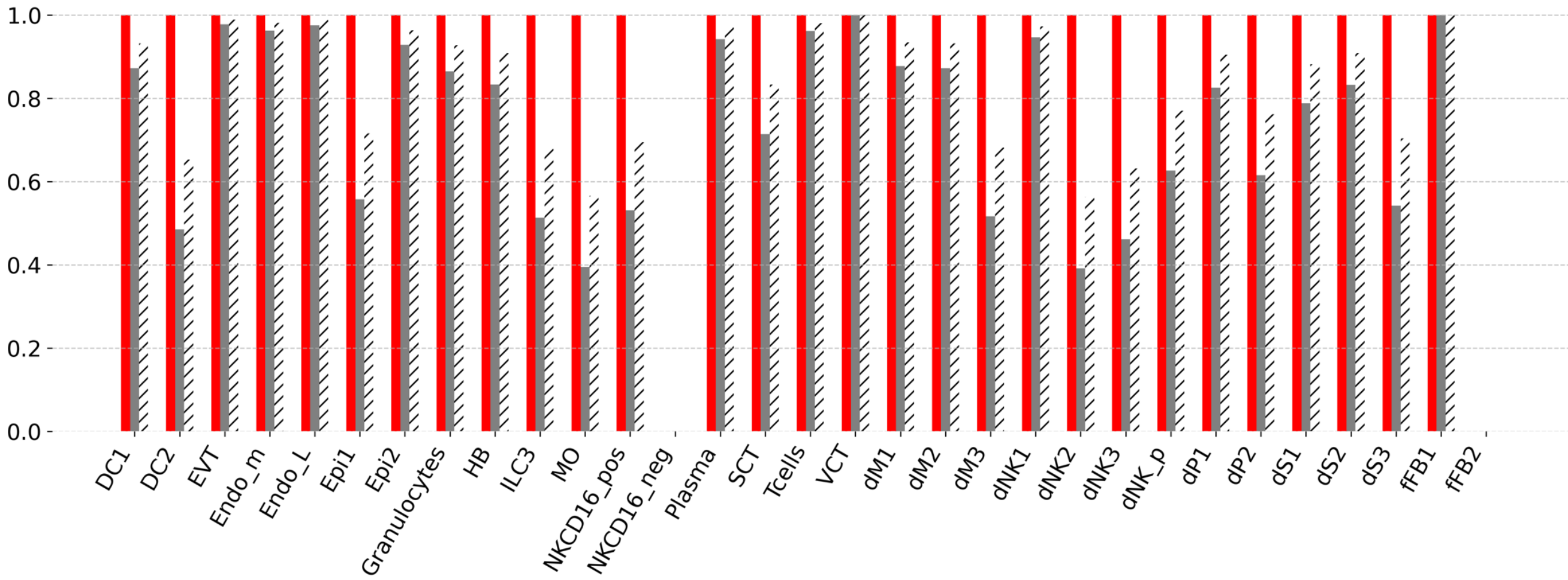

B.

<Du's dataset>

2nd level

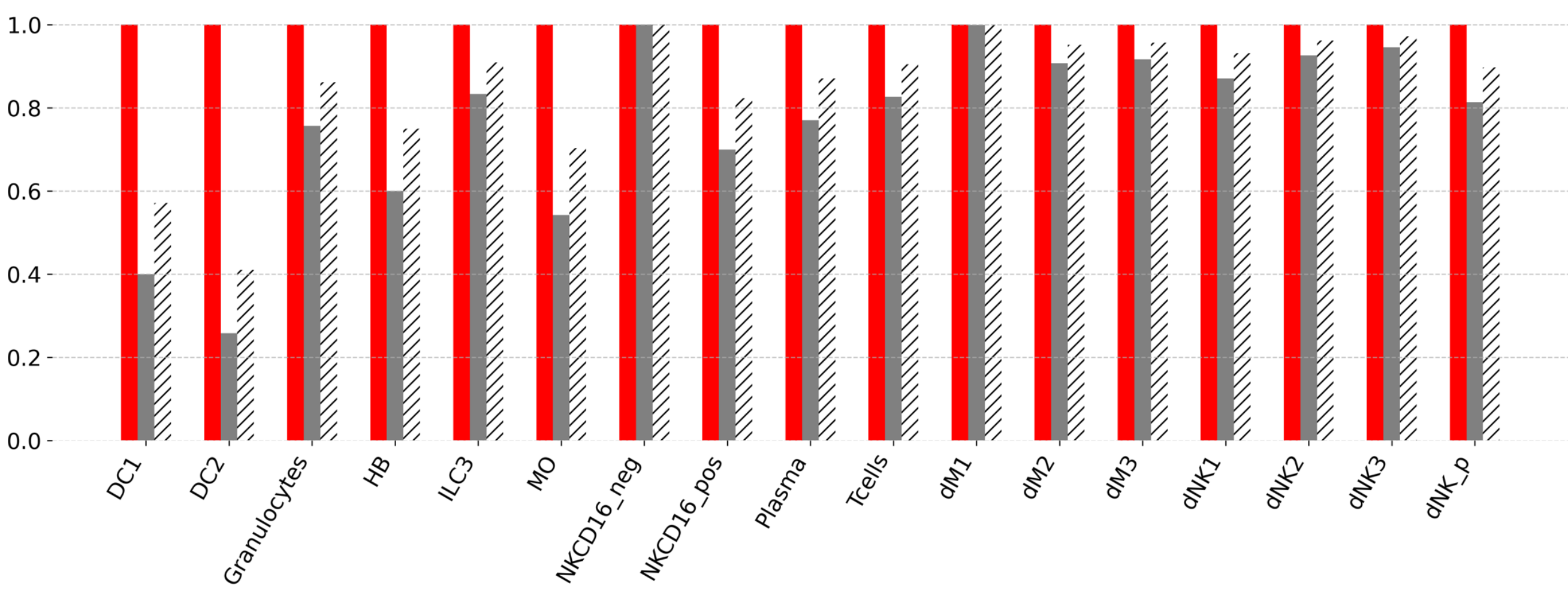

C.

<Vento's dataset>

1st level

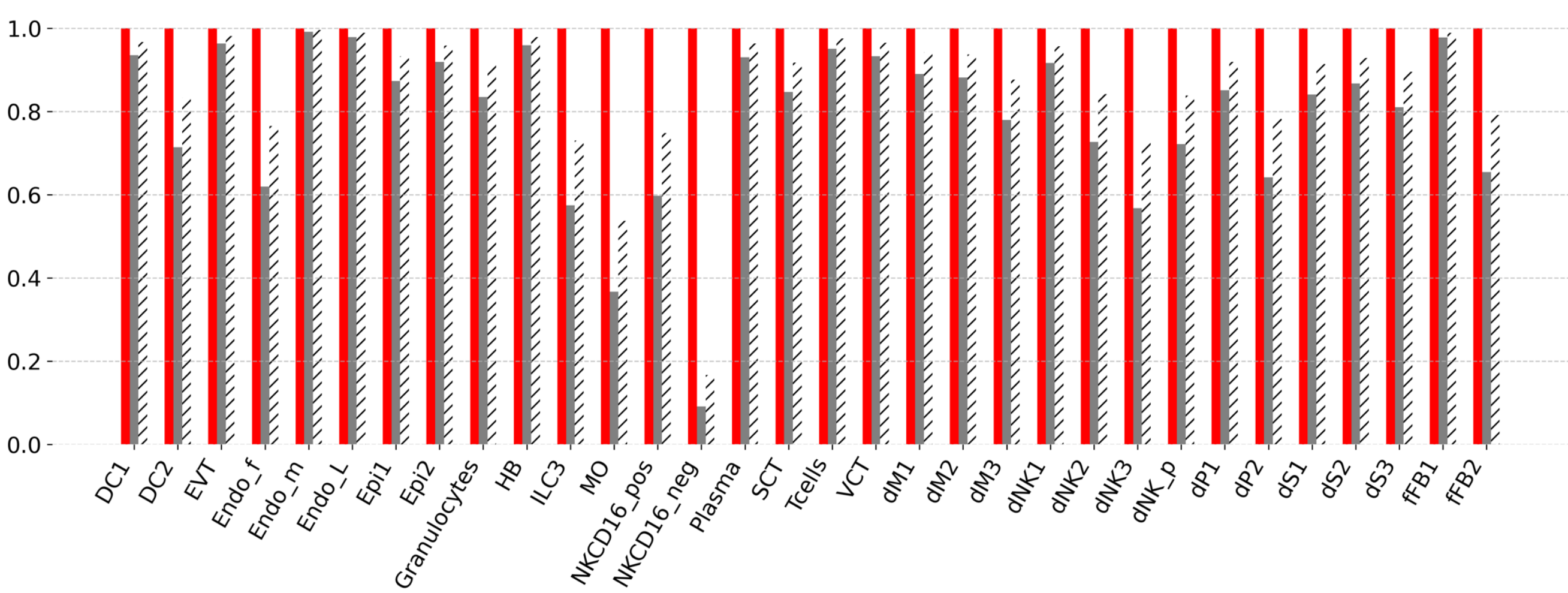

Supplement: Supplementary file 5 [file pcbi.1014806.s005.pdf]
